# Deep Learning‐Enhanced Hand‐Driven Microfluidic Chip for Multiplexed Nucleic Acid Detection Based on RPA/CRISPR

**DOI:** 10.1002/advs.202414918

**Published:** 2025-03-31

**Authors:** Tao Xu, Ying Zhang, Shunji Li, Chenxi Dai, Hongguo Wei, Dongjuan Chen, Yunjun Zhao, He Liu, Deliang Li, Peng Chen, Bi‐Feng Liu, Ye Tian

**Affiliations:** ^1^ College of Medicine and Biological Information Engineering Northeastern University Shenyang 110169 China; ^2^ The Key Laboratory for Biomedical Photonics of MOE at Wuhan National Laboratory for Optoelectronics‐Hubei Bioinformatics & Molecular Imaging Key Laboratory Systems Biology Theme Department of Biomedical Engineering College of Life Science and Technology Huazhong University of Science and Technology Wuhan 430074 China; ^3^ Department of Laboratory Medicine Maternal and Child Health Hospital of Hubei Province Tongji Medical College Huazhong University of Science and Technology Wuhan 430070 China; ^4^ Foshan Graduate School of Innovation Northeastern University Foshan 528300 China

**Keywords:** artificial intelligence, community screening, CRISPR, hand‐driven microfluidic, high‐risk human papillomavirus

## Abstract

The early detection of high‐risk human papillomavirus (HR‐HPV) is crucial for the assessment and improvement of prognosis in cervical cancer. However, existing PCR‐based screening methods suffer from inadequate accessibility, which dampens the enthusiasm for screening among grassroots populations, especially in resource‐limited areas, and contributes to the persistently high mortality rate of cervical cancer. Here, a portable system is proposed for multiplexed nucleic acid detection, termed **R‐CHIP**, that integrates **R**ecombinase polymerase amplification (RPA), **C**RISPR detection, **H**and‐driven microfluidics, and an artificial **I**ntelligence **P**latform. The system can go from sample pre‐processing to results readout in less than an hour with simple manual operation. Optimized for sensitivity of 10^−17^ M for HPV‐16 and 10^−18^ M for HPV‐18, R‐CHIP has an accuracy of over 95% in 300 tests on clinical samples. In addition, a smartphone microimaging system combined with the ResNet‐18 deep learning model is used to improve the readout efficiency and convenience of the detection system, with initial prediction accuracies of 96.0% and 98.0% for HPV‐16 and HPV‐18, respectively. R‐CHIP, as a user‐friendly and intelligent detection platform, has great potential for community‐level HR‐HPV screening in resource‐constrained settings, and contributes to the prevention and early diagnosis of other diseases.

## Graphical abstract

**Efficient R‐CHIP HR‐HPV Screening System**: The R‐CHIP system utilizes the RPA/CRISPR method, a hand‐driven centrifugal microfluidic device, a smartphone micro‐imaging system, and the ResNet‐18 deep‐learning algorithm to simplify the sample detection process, ensure accurate results, and reduce costs. The system screens for multiple high‐risk HPV infections within one hour, making it ideal for the community and resource‐limited areas.

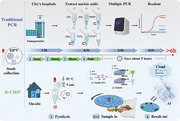

## 1 Introduction

As we all know, HR‐HPV‐related diseases are generally treatable, While patient prognosis heavily relies on the timing of treatment. Lack of portable screening technology is one of the key to the global incidence of HPV‐related cancers has steadily increased, with cervical cancer being particularly severe, accounting for over 600000 new cases annually, 80% of which occur in low‐ and middle‐income countries (LMICs).^[^ ^1^, ^2^ ^]^ Persistent infection with high‐risk human papillomavirus (HR‐HPV) is recognized as the primary cause of cervical cancer, and if not diagnosed and treated early, it can progress to fatal diseases such as cervical, anal, and head and neck cancers, posing a significant threat to women's health worldwide.^[^ ^3^, ^4^ ^]^ Notably, the HPV‐16 and HPV‐18 genotypes play a central role in cervical cancer and its precursors, accounting for approximately 70% of related cases globally.^[^ ^5^, ^6^ ^]^ Early diagnosis and intervention are crucial for improving survival rates, especially in the precancerous stages.^[^ ^7^, ^8^ ^]^ Therefore, HR‐HPV screening has been established as a core intervention for the prevention of cervical cancer.

Nonetheless, the current PCR‐based screening methods for HR‐HPV lack convenience and are time‐consuming. Polymerase chain reaction (PCR) technology is widely used for the detection of HPV DNA/mRNA in various biological samples (including cervical smear, anal and oral swab or saliva) due to its high sensitivity and specificity, with multiplex PCR being particularly favored for its ability to screen multiple HPV types simultaneously.^[^ ^9^, ^10^ ^]^ However, limitations such as the need for specialized laboratory settings, complex instrumentation, precise temperature control, high costs, and specialized personnel restrict PCR techniques to large clinical laboratories. This results in a feedback cycle of 1–2 days for test results, inadvertently increasing the additional medical burdens on patients and affecting HPV screening participation, especially in resource‐limited regions.^[^ ^10^, ^11^, ^12^, ^13^, ^14^ ^]^ Research indicates that the accuracy of HPV detection from self‐collected cervical samples is comparable to that from clinician‐collected samples. Moreover, self‐collection can overcome barriers related to healthcare resources and privacy, demonstrating significant potential for community screening in low‐ and middle‐income countries.^[^ ^15^, ^16^, ^17^ ^]^ In light of this, the development of a facile and efficient HR‐HPV community screening methodology holds paramount significance in enhancing the accessibility and prevalence of HPV detection, thereby eradicating geographical and socio‐economic barriers to cervical cancer screening and propelling global efforts towards cervical cancer prevention and control.

To enhance the convenience of HPV screening and reduce the time for sample processing and result interpretation, various techniques have emerged, including isothermal amplification, immunoblotting, lateral flow immunoassays, electrochemical detection, molecular sequencing, and CRISPR‐based methods.^[^ ^11^, ^18^, ^19^, ^20^, ^21^ ^]^ Among these, CRISPR‐Dx has gained significant attention in the field of biosensing due to its efficiency, simplicity, high specificity, and sensitivity.^[^ ^22^, ^23^, ^24^, ^25^, ^26^ ^]^ The SHERLOCK platform, the first application of CRISPR‐Dx, utilizes Cas13a combined with reverse transcription recombinase polymerase amplification (RT‐RPA) for the successful detection of Zika and dengue viruses.^[^ ^27^, ^28^ ^]^ Subsequently, Wang et al. demonstrated that Cas12a possesses RNA‐guided DNA specificity and developed the HOLMES DNA detection technology based on this capability.^[^ ^29^, ^30^ ^]^ The Doudna team further introduced the “DETECTR” platform using CRISPR‐Cas12a, enabling rapid and specific detection of HPV‐16/18 in clinical samples.^[^ ^31^ ^]^ However, single CRISPR‐Dx technologies often struggle to simultaneously meet the demands for convenience and high sensitivity in practical applications.^[^ ^32^, ^33^, ^34^, ^35^ ^]^ Microfluidic technology, with its precise fluid control, efficient sample processing capabilities, automation features, and low sample requirements, has emerged as an ideal choice for integrating various detection methods.^[^ ^36^, ^37^, ^38^, ^39^, ^40^ ^]^ Xu et al. proposed a nucleic acid testing platform named MiCaR, which integrates a starburst‐shaped microchip (SS‐Chip) with CRISPR‐Cas12a and multiplex recombinase polymerase amplification to effectively detect nine HPV subtypes.^[^ ^41^ ^]^ Lee developed a point‐of‐care nucleic acid testing platform called CreDiT, which combines a portable instrument and self‐developed algorithms to achieve effective diagnosis of multiple HPV subtypes.^[^ ^42^ ^]^ Despite the significant potential of microfluidic technology in HPV screening, its application in community settings faces numerous challenges. These challenges primarily stem from the reliance on complex and bulky equipment for fluid control, which requires skilled operators and stringent quality control.^[^ ^43^ ^]^ Moreover, the seamless integration of the entire HPV detection process sample preprocessing, amplification, and detection on a microchip necessitates precise and sequential release of reagents, followed by their high‐throughput allocation to individual reaction chambers for efficient mixing. Currently, integrating sample preprocessing, automated on‐demand reagent release, high‐throughput quantitative sampling, and independent, parallel high‐throughput mixing reaction units on a single chip remains a significant challenge.^[^ ^44^ ^]^

**Figure 1:**
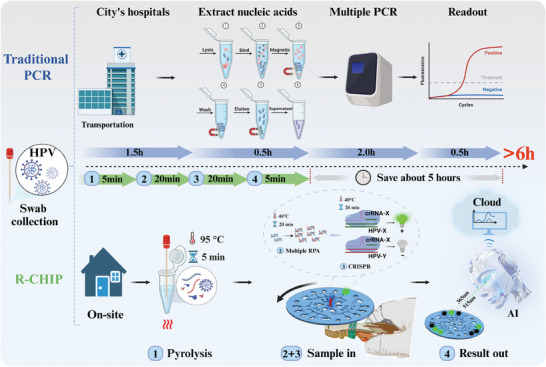
Flowchart of HR‐HPV screening using the R‐CHIP microfluidic chip. The R‐CHIP screening process includes sample heat lysis, RPA amplification, and CRISPR cleavage detection. Traditional PCR detection usually takes more than 6 hours to report results (top). In comparison, R‐CHIP can provide results within 1 hour (bottom). Some graphical elements were created using biorender.com.

Centrifugal microfluidic technology utilizes the centrifugal, Coriolis, and Euler forces generated by the rotation of a disk to achieve precise manipulation of samples and reagents. This technology offers potential for fully automated detection, low cross‐contamination risk, no need for external pumping systems, and efficient point‐of‐care (POC) testing, garnering significant attention in the field.^[^ ^45^, ^46^, ^47^ ^]^ Recently, our team has developed a series of centrifugal microfluidic technologies based on passive and active valves, successfully applied in antibiotic sensitivity assessment, immunoassays, nucleic acid detection, and phase separation.^[^ ^48^, ^49^, ^50^, ^51^ ^]^ Although these chips demonstrate good stability and compatibility with various reagents, their operation involves complex mechanical control processes. Here, we introduce R‐CHIP, which features a clever design of active and passive valves to achieve sample preprocessing and on‐demand quantitative release of reagents under low‐speed manual operation, meeting the needs of HPV community screening. R‐CHIP integrates manual centrifugation, isothermal amplification, and reagent distribution functionalities, and is compatible with household heating devices and miniaturized instruments, showcasing exceptional convenience, multifunctionality, and programmability. By incorporating CRISPR technology, the R‐CHIP not only overcomes the issue of aerosol contamination caused by repeated lid opening and liquid transfer in traditional two‐step methods but also addresses the limitations of One‐pot methods, such as inadequate specificity, stability, reaction rate, and sensitivity.^[^ ^52^ ^]^ By utilizing a smartphone micro‐imaging system combined with deep learning for image recognition, R‐CHIP can rapidly detect HR‐HPV subtypes in multiple samples simultaneously on a single chip, eliminating the need for multiple fluorescent labels and significantly improving detection efficiency and accessibility. Experimental results indicate that R‐CHIP achieves detection sensitivities of 1 × 10^−17^M and 1 × 10^−18^ M for HPV‐16 and HPV‐18 amplified plasmids, respectively, and the results of clinical samples were highly consistent with multiplex PCR. In summary, R‐CHIP, with its user‐friendly design, robust applicability, flexible configuration, and significant cost‐effectiveness, is poised to become a powerful tool for point‐of‐care diagnosis in HPV screening and various biomarker detections. It is expected to play a crucial role in early cancer warning, rapid response to infectious diseases, home health self‐testing, and large‐scale population screening, driving innovation and advancement in medical diagnostic technology (**Figure** **1**).

## 2 Results and Discussion

### 2.1 The Structure and Principle of R‐CHIP

**Figure 2:**
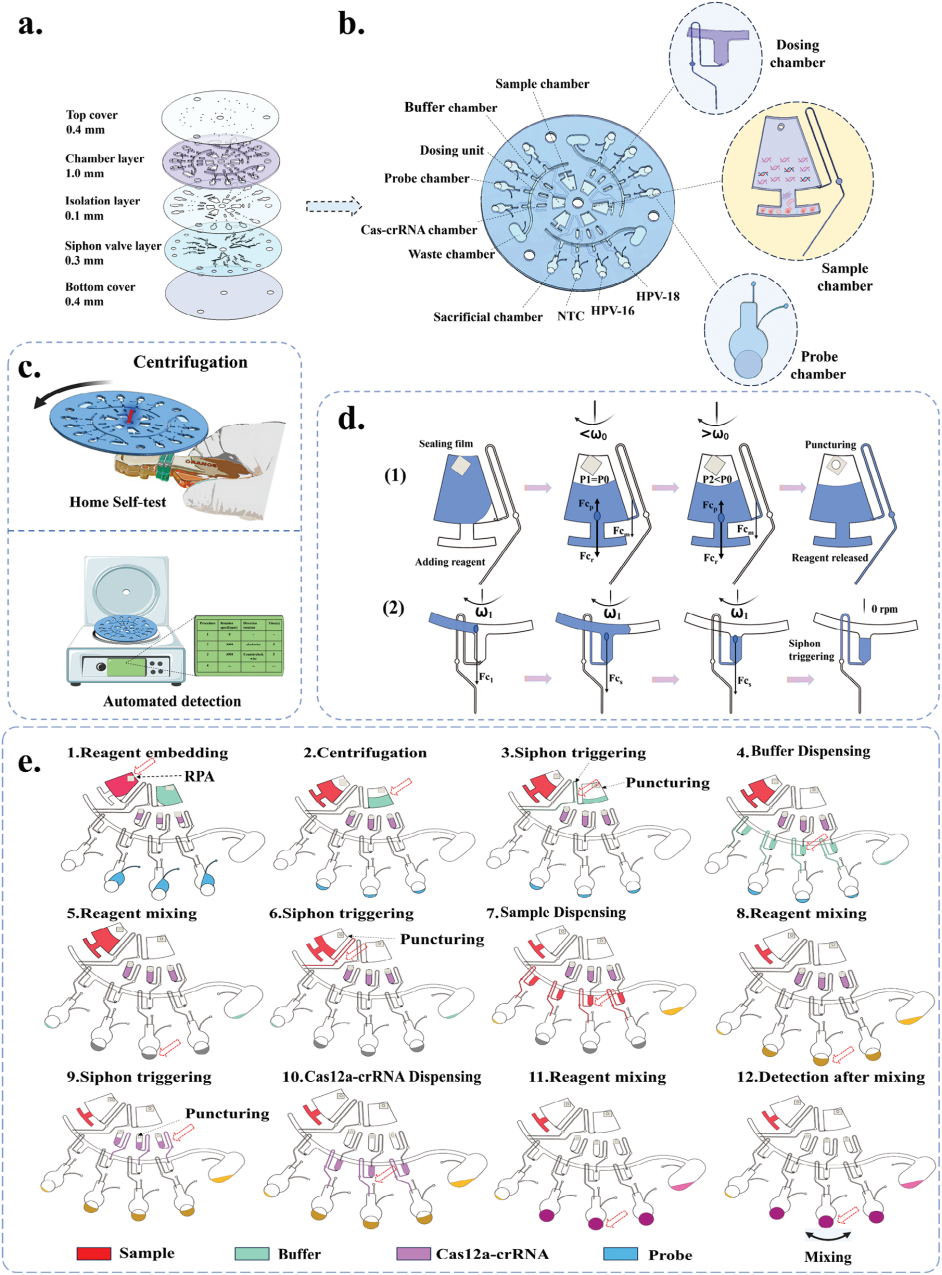
Structure and detection process of R‐CHIP. a) Schematic representation of the layered structure of the R‐CHIP chip. b) Distribution map of the various chambers within the R‐CHIP chip. c) Hand‐driven and automated centrifugation programs for R‐CHIP chip. d) Force analysis of the R‐CHIP structural components. e) Flowchart illustrating the embedding of reagents and the reagent release process in R‐CHIP.

In this study, an integrated centrifugal microfluidic detection system, R‐CHIP, was developed. This system innovatively combines handheld convenience with efficient sample processing capabilities, integrating sample amplification, sequential reagent release, and CRISPR detection technologies, making it particularly suitable for community screening of HR‐HPV. The R‐CHIP chip consists of five structural layers: a top cover (0.4 mm), a chamber layer (1.0 mm), an isolation layer (0.1 mm), a siphon valve layer (0.3 mm), and a bottom cover (0.4 mm). It incorporates three parallel working units, each equipped with a complete set of detection components, including Sample chamber, Buffer chamber, Cas‐crRNA chamber, Dosing unit, Probe chambers, and waste chamber. The Sample chamber encapsulates RPA amplification reagents for amplification and separation of pyrolysis samples. The Buffer chamber provides the necessary buffering environment, containing NEB buffer. The Cas‐crRNA chamber pre‐complexes the Cas12a protein with crRNA, thereby providing some protection against crRNA degradation. The Dosing unit includes liquid distribution channels, a waste chamber, and four Dosing chamber, ensuring precise allocation of reaction components. The probe chamber contains TBA11 and includes a sacrificial chamber, a negative control chamber (NTC), and reaction chambers for HPV‐16 and HPV‐18, facilitating reagent mixing and fluorescence signal detection (In the probe chamber, TBA11 can be replaced with other fluorescent probes. TBA11, a shortened version of Thrombin‐Binding Aptamer (TBA) labeled with a fluorescence quencher (FQ), has been shown to exhibit higher sensitivity compared to traditional single‐stranded DNA (ssDNA) reporters.). The sacrificial chamber is designed to accommodate residual reagents to prevent contamination. The circular detection port of the probe chamber features a unique penetration design, directly piercing through the siphonic valve layer to connect to the bottom cover, preventing reagent backflow (**Figure** **2**a,b). After pre‐loading the reagents, the inlets of each chamber are sealed with a film. During the detection process, the reagents are sequentially released by centrifugally puncturing the film. The specific operating steps are as follows:

To ensure the stability and activity of each component, the RPA amplification reagents, NEB buffer, Cas12a‐crRNA complex, and TBA11 fluorescent probe are individually encapsulated in dedicated chambers to prevent deactivation due to premature mixing. The pyrolyzed sample was then added to the sample amplification chamber, sealed, and subjected to amplification and centrifugation. Multiple cycles of centrifugation remove CRISPR interferents such as cellular debris and concurrently activate the syphon valves in each chamber (Figure 2c). Once activated, the films are punctured sequentially and hand centrifuged for 10 seconds to release the NEB buffer, amplified samples, and Cas12a‐crRNA mixture into the probe chamber. After a 20‐minute incubation, the Cas12a‐crRNA recognizes the amplified target sequences, activating its ssDNA trans‐cleavage activity to cleave the TBA11 fluorescent quencher probes, thereby releasing fluorescence. Finally, fluorescence values of the corresponding negative control (NTC), HPV‐16, and HPV‐18 assay wells were measured using a fluorescence microscope or smart microimaging system to assess the type of infection in each sample (Figure 2e).

### 2.2 Mechanical Feasibility Evaluation of R‐CHIP

Sequentially release principle: As shown in Figure 2d (1), the direct connection between the reagent chamber and the external atmosphere is effectively sealed after the reagents are preloaded and the sample ports are closed using a one‐sided pressure‐sensitive adhesive sealing film. Within this sealed system, the gas pressure follows the ideal gas law equation:

(1) PV=NRT

where *P* is pressure, *V* is volume, *N* is the mole number of the gas, *R* is the gas constant, and *T* is temperature.

Under the centrifugal action, the liquid is driven into the siphon valve channel by *Fc_r_*, which causes the remaining volume in the sealed chamber to expand, leading to a decrease in the internal gas pressure and the creation of a vacuum suction force *Fc_p_* on the liquid, as follows:

(2) $$Fc_{p}=Kv\Delta P$$

(3) $$\Delta P=P_{0}-P_{x}$$

*Fc_p_* is the suction force exerted by the pressure difference on the unit volume of the liquid, where *K* is a constant under fixed conditions, ***v*** is the unit volume of the solution, *P_0_* is the atmospheric pressure, and *P_x_* is the air pressure inside the chamber, with *ΔP* being the pressure difference.

Additionally, due to the hydrophilic modification of the siphon valve channel, the liquid in the channel is also subject to the capillary force *Fc_m_*:^[^ ^53^ ^]^

(4) $$Fc_{m}=P_{m}S$$

(5) $$p_{m}=-\gamma \left[\frac{\cos\theta_{t}+os\theta_{b}}{h}+\frac{\cos\theta_{l}+os\theta_{r}}{w}\right]$$

where *Fc_m_* is the capillary force, *P_m_* is the capillary pressure, and *S* is the unit area of the solution; *γ* is the surface tension of the liquid in the microchannel, *h* and *w* are the height and width of the channel, and *θ_t_, θ_b_, θ_l_*, and *θ_r_* are the contact angles of the liquid with the corresponding four microchannel walls.

Integrating the above mechanical analysis, the total force on the liquid must satisfy the balance condition of *Fc_r_* + *Fc_m_ = Fc_p_*. In this scenario, *Fc_r_* and *Fc_m_* act in the same direction, and the sealed chamber's design parameters set a critical rotational speed *w_0_*. Above this speed, although the chamber's internal pressure decreases (*P_0_>P_2_*), the radial decreasing characteristic of the centrifugal force ensures that the liquid level in the siphon valve channel does not get closer to the center than the chamber, thereby avoiding unintended triggering. Conversely, if the rotational speed is lower than *w_0_* or stopped, the combined force of *Fc_r_* and *Fc_m_* is too weak to significantly change the volume or trigger the siphon valve. However, once the sealing film is punctured and the chamber's negative pressure is released, *Fc_p_* becomes 0, and the system is then solely dominated by *Fc_m_*, which promptly fills the siphon valve channel and triggers its functionality.

**Dispensing module**: As shown in Figure 2d (2), When the solution in the reagent chamber triggers the siphon valve, the solution can be rapidly transported to the downstream metering chamber by increasing the centrifugal velocity *w_1_* and acceleration. In this process, the solution enters the metering chamber under the action of the centrifugal force, and it preferentially flows along the path of the maximum centrifugal force to ensure rapid and effective solution distribution, following the formula:

(6) $$Pc_{1}=\frac{\rho \times \omega_{1}^{2}\times \left(r_{0}^{2}-r_{i}^{2}\right)}{2}$$

here *ρ* is the density of the liquid; *ω* is the rotation speed of the centrifugal microfluidic chip; *r_0_* is the maximum radius of the liquid region; *r_i_* is the minimum radius of the liquid region.

Since the valve is in the critical state of bursting, the liquid is not subjected to flow resistance, and the surface tension and centrifugal force are balanced, i.e., *Pc*_1_ = *p_m_* Thus, by relating the relevant equations, the formula for solving for the bursting speed is obtained, which is as follows:

(7) $$\omega_{1}=\sqrt{-\frac{2\gamma \left(\frac{\cos\theta_{t}+os\theta_{b}}{h}+\frac{\cos\theta_{t}+os\theta_{r}}{w}\right)}{\rho \times \left(r_{0}^{2}-r_{i}^{2}\right)}}$$

Furthermore, to achieve precise metering, this module is cleverly designed with the dosing chamber and the reaction chamber (probe chamber) connected by a siphon channel, with its highest point carefully positioned closer to the rotational center. This design ensures that at high centrifugal speeds and high accelerations, the siphon valve can be maintained in a closed state, and any excess solution is automatically directed through the dosing distribution unit to the next dosing chamber or waste chamber, thereby ensuring the accuracy of liquid aliquoting. The relevant formula is as follows:

(8) $$Fc_{s}=m\omega_{1}^{2}r$$

It is worth noting that when the centrifugal speed is reduced below a certain threshold or completely stopped, the capillary force will dominate the behavior of the solution, automatically filling the siphon valve channel and preparing for the subsequent operation. Through systematic experimental studies, we have determined that under conditions where the final centrifugation speed exceeds 2000 rpm and the acceleration is no less than 500 rpm ^−1^s, the relevant detection process can be stably and reliably performed through a single repetitive step (Figure S1a–e, Supporting Information).

To evaluate the usability of the device across different populations, we randomly recruited 30 volunteers from diverse backgrounds for testing. The experimental results indicate that the vast majority of participants were able to successfully achieve the required final speed and acceleration standards, demonstrating that the hand‐driven portable device can relatively easily meet the specified operational requirements (Figure S1f, Supporting Information). To further validate its applicability, we randomly selected 11 female volunteers to participate in the complete R‐CHIP detection process. The results show that all participants were able to successfully complete each step of the operation (Figures S2,S3, Supporting Information). These findings further confirm that the system design is not only practical but also highly reliable, showcasing the stability and convenience of the device across a broad user base.

### 2.3 Optimization of the CRISPR Detection System

**Figure 3:**
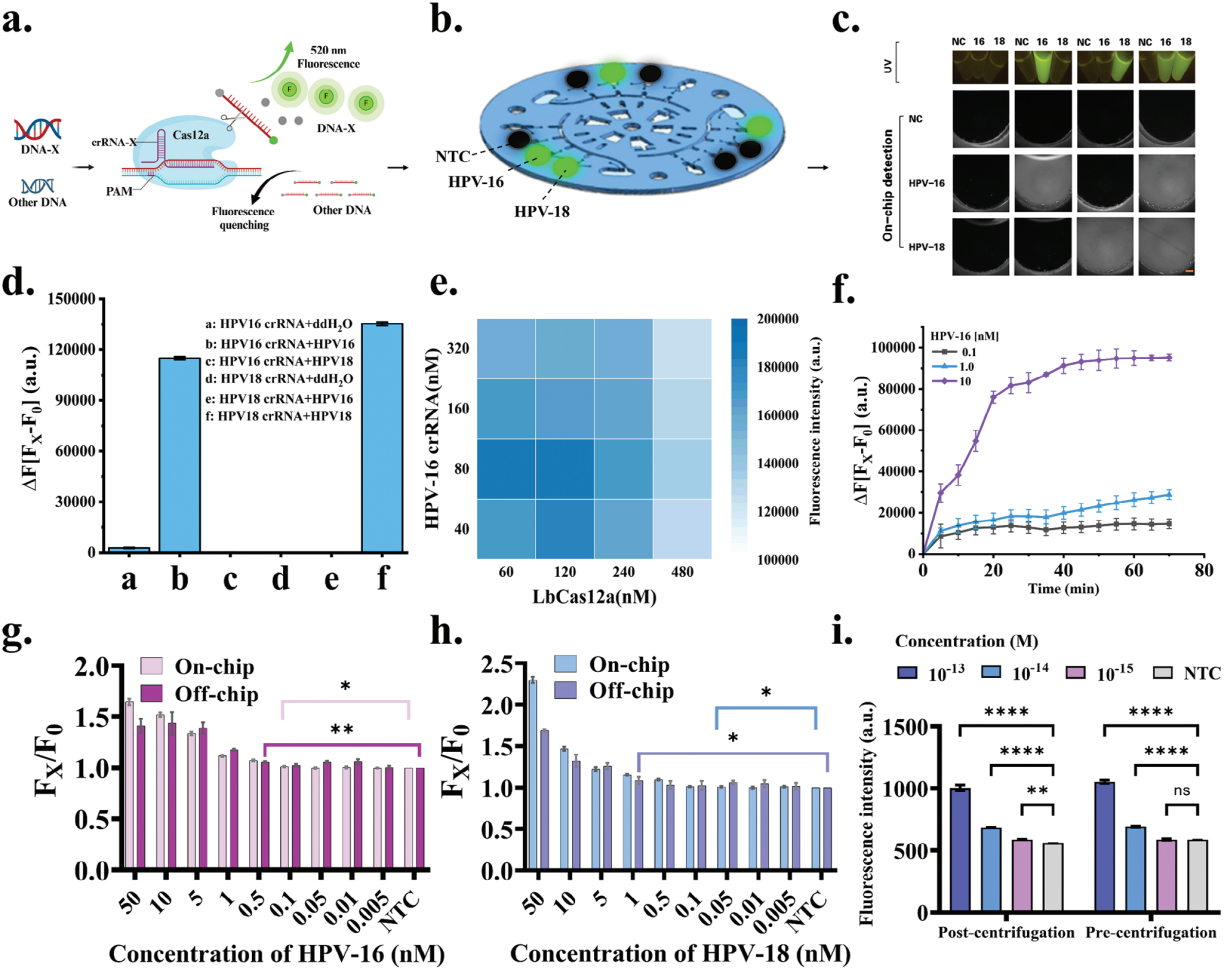
Detection protocol for HR‐HPV and On‐chip feasibility validation. a) Schematic representation of the CRISPR detection process. b) Display of positive and negative results from the R‐CHIP. c) Illustration differentiating positive and negative HR‐HPV detections inside and outside the R‐CHIP. d) Specificity validation of the Cas12a‐crRNA system. n = 3. e) Optimization of Cas12a and crRNA concentrations within the CRISPR detection system. n = 3. f) Determination of the optimal detection time for the CRISPR system. n = 3. g, h) Comparison of non‐amplification sensitivity for detection of HPV‐16 and HPV‐18 plasmids n and off‐chip. n = 3. i) The effect of hand‐driven centrifugation on CRISPR detection results after amplification of standard HPV cell lysates at different concentrations. n = 3. Significance indicated: *P‐value < 0.05, **P‐value < 0.01, ***P‐value < 0.001, ****P‐value < 0.0001. Scale bar: 1cm.

In the CRISPR detection system, Cas12a protein and crRNA serve as crucial components, responsible for both target recognition and signal transduction (**Figure** **3**a–c). Therefore, the ssDNA fluorescent quenching probe TBA11 was first used to verify the specific DNA endonuclease activity and the associated non‐specific ssDNA enzyme activity of Cas12a. HPV‐16, HPV‐18 plasmids and ddH_2_O were employed as target analytes, with corresponding crRNAs designed. The experimental system sequentially added NEB buffer, Cas12a protein, crRNA, TBA11, ddH_2_O, and the target analyte, followed by incubation at 37 °C for 30 minutes. Fluorescence readings were taken using a full‐wavelength microplate reader (excitation: 483 ± 14 nm; emission: 530 ± 30 nm). Only when Cas12a‐crRNA recognizes the corresponding target DNA (HPV‐16 or HPV‐18 DNA, but not ddH_2_O) is TBA11 hydrolyzed, releasing fluorescence (Figure 3d). This confirms the target sequence specificity of Cas12a‐crRNA and the activation of its collateral nonspecific ssDNA nuclease activity, leading to probe cleavage. To ascertain the optimal concentrations of Cas12a protein and crRNA, a concentration optimization of Cas12a and its corresponding crRNA within the reaction system was conducted using the HPV‐16 plasmid. Fluorescence intensities were measured on a full‐wavelength microplate reader, revealing peak fluorescence intensity when the final concentrations of Cas12a and crRNA were set at 60 nM, 120 nM, and 80 nM, respectively (Figure 3e). With the aim of conserving Cas12a protein usage, the final concentrations of 60 nM for Cas12a and 80 nM for crRNA were selected for subsequent experiments.

Finally, the optimal detection time of the reaction system was optimized using different concentrations of HPV‐16. To determine the optimal detection time, fluorescence intensity values were collected at different time points from various samples. The results showed that the relative fluorescence signals of positive test samples increased gradually over time and stabilized after 20 minutes (Figure 3f). Considering the duration of the detection and signal strength, 20 minutes was selected as the optimal readout time for the detection system.

### 2.4 The Verification of R‐CHIP chip

This study first established HPV‐16 and HPV‐18 plasmid samples at different concentrations and preliminarily evaluated the non‐amplified sensitivity of the CRISPR detection system Off‐chip using centrifuge tubes. The results showed that the non‐amplified sensitivities of HPV‐16 and HPV‐18 Off‐chip were 0.5 nM and 1 nM, respectively (Figure S4, Supporting Information). Subsequently, the non‐amplified sensitivities of HPV‐16 and HPV‐18 were tested On‐chip using the CRISPR detection system. The results demonstrated a significant improvement in sensitivity On‐chip, with sensitivities of 0.1 nM and 0.05 nM for HPV‐16 and HPV‐18, respectively (Figure 3g,h). The increased On‐chip sensitivity may be attributed to the lower fluorescence intensity of the low‐concentration samples Off‐chip and greater environmental interference, making it difficult for the analysis software to distinguish them from negative controls. In further experiments, standard HPV‐18 cells with different plasmid concentrations were used to evaluate the effect of cell lysates before and after R‐CHIP manual centrifugation on CRISPR assay results. The results showed that the fluorescence intensity values were reduced after hand‐driven centrifugation compared to no centrifugation, but the sensitivity after hand‐driven centrifugation was higher than that of CRISPR detection without centrifugation. This suggests that certain interferents in the cell lysate may affect the cleavage of quenched probes, and these interferents are reduced after hand‐driven centrifugation (Figure 3i). These findings highlight the potential of R‐CHIP in CRISPR detection and sample preprocessing.

### 2.5 Design of Multiplex Primers for HPV‐16 and HPV‐18

**Figure 4:**
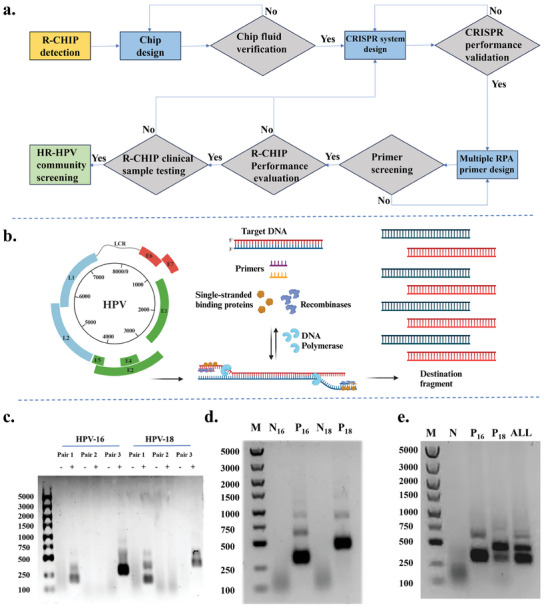
Multiplex RPA assay design for HR‐HPV detection. a) Design Flowchart of R‐CHIP. b) Schematic illustration of the RPA amplification principle. c) Optimization of primers for RPA amplification. d) Singleplex RPA amplification of HR‐HPV (P16∖P18 and N16∖N18 represent bands amplified with and without the addition of HPV‐16∖18 plasmids, respectively). e) Multiplex RPA amplification of HR‐HPV (N denotes the addition of H_2_O as a Negative control, P16∖P18 as described in Figure 4d, and “ALL” indicates the multiplex RPA amplification with simultaneous addition of HPV16, 18 plasmids, and primers).

To enhance the HR‐HPV detection capability of the R‐CHIP platform (**Figure** **4**a), this study introduced RPA isothermal amplification technology and designed specific forward and reverse primers targeting the unique genomic regions of HPV‐16 and HPV‐18 (Figure 4b and Table S1, Supporting Information). Various primer pairs were evaluated for their sensitivity and specificity in amplifying the corresponding HPV‐16 and HPV‐18 target regions, with primer pair 3 demonstrating the best performance (Figure 4c). Subsequently, these optimized primers were utilized for both singleplex and multiplex RPA amplification of HPV‐16 and HPV‐18(Figure 4d,e). The experimental results demonstrated that the optimized primer pairs exhibited good sensitivity and specificity, effectively facilitating multiplex isothermal amplification of HPV‐16 and HPV‐18 subtypes, thereby significantly improving the detection capability for clinical samples.

### 2.6 Evaluation of R‐CHIP

**Figure 5:**
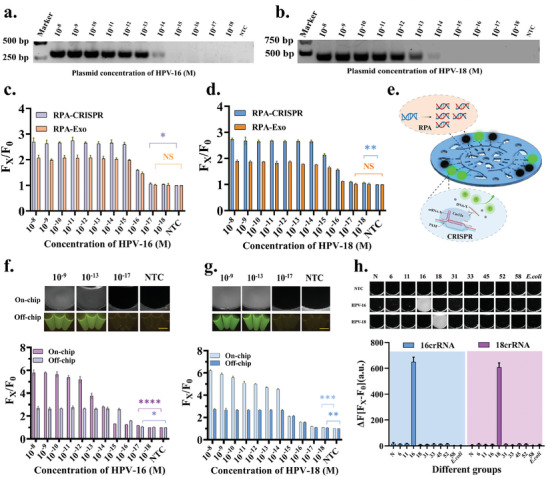
Evaluation of R‐CHIP's Comprehensive Performance. a, b) Gel electrophoresis images of HPV‐16 and HPV‐18 plasmids after RPA amplification. c, d) Off‐chip sensitivity comparison for HPV‐16 and HPV‐18: RPA‐CRISPR method versus RPA fluorescence method. n = 3. e) Schematic of On‐chip CRISPR detection following RPA amplification. f, g) On‐chip and Off‐chip detection images and sensitivity analysis for HPV‐16 and HPV‐18 plasmids using the RPA‐CRISPR method. n = 3. h) On‐chip specific detection results of multiple pathogens under the HR‐HPV detection system, including CCD images and bar graph analysis. n = 3. Significance indicated: *P‐value < 0.05, **P‐value < 0.01, ***P‐value < 0.001, ****P‐value < 0.0001. Scale bar: 1 cm.

To validate the HPV detection capability of R‐CHIP, The experiment first assessed the sensitivity of RPA isothermal amplification. Gel electrophoresis analysis of varying concentrations of HPV‐16 and HPV‐18 plasmids revealed a detection limit as low as 10^−14^ M, demonstrating the high sensitivity of RPA amplification (**Figure** **5**a,b). Further comparing the differences between the sensitivities of the methods, the detection RPA system was designed and the Exo probe was optimized to explore the RPA fluorescence method for HR‐HPV detection. The results showed that the detection limit of the RPA fluorescence method for HPV‐16 and HPV‐18 plasmids could be further reduced to 10^−16^ M (Figure S5, Supporting Information), highlighting the potential of the RPA Exo probe in enhancing the sensitivity of HR‐HPV detection. To further evaluate the sensitivity difference between RPA Exo and RPA‐CRISPR methods, a series of constructs containing different concentrations of HPV plasmids were prepared. The experimental results showed that the detection limits of HPV‐16 and HPV‐18 plasmids were further reduced to 10^−18^ M after the introduction of the two signal amplification steps of RPA‐CRISPR in the R‐CHIP system (Figure 5c,d). This demonstrates that our detection system is extremely sensitive and the detection limit is several orders of magnitude lower than that of the conventional RPA fluorescence method.

Multiplexed RPA amplification and CRISPR reactions were performed using a microfluidic chip in order to fully evaluate the comprehensive performance of the R‐CHIP detection system (Figure 5e). Quantitative analysis by fluorescence microscopy showed that the detection limits of HPV‐16 and HPV‐18 were 10^−17^M and 10^−18^M, respectively (Figure S6, Supporting Information). And the difference in sensitivity between Off‐chip sensitivity and On‐chip detection was minimal (Figure 5f,g), and the results were stable (Figure S7,S8, Supporting Information). To validate the specificity of this detection system, we applied the multiplex RPA‐CRISPR detection system to the gene sequence detection of all subtypes covered by the HPV nine‐valent vaccine and the common vaginal pathogen *E. coli*. Initially, our experiment compared the L1 gene sequences of nine high‐risk HPV types (including HPV‐6, HPV‐11, HPV‐16, HPV‐18, HPV‐31, HPV‐33, HPV‐45, HPV‐52, and HPV‐58). Different colors were used to highlight conserved regions among these subtypes, and sequence alignment clearly demonstrated the genetic variation characteristics among different HPV subtype L1 gene sequences, as well as the amplification sites and crRNA‐specific recognition sites for HPV‐16 and HPV‐18 (Figure S9, Supporting Information). Preliminary gel electrophoresis results indicated cross‐reactivity of the HR‐HPV multiplex RPA detection system with multiple HPV subtypes (Figure S10a, Supporting Information). However, the introduction of the CRISPR cleavage step effectively eliminated non‐specific signals and ensured high specificity of the detection (Figure 5h; Figure S10, Supporting Information). These findings further confirm the high sensitivity, specificity, and stability of the R‐CHIP in detecting HPV viruses.

### 2.7 Clinical HPV Sample Detection Based on R‐CHIP Strategy

**Figure 6:**
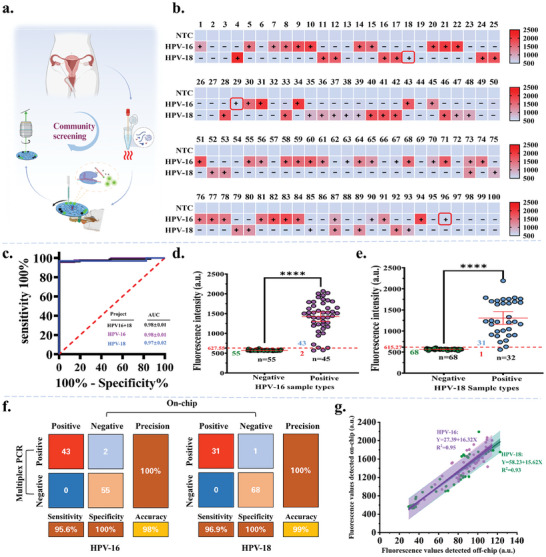
Clinical Sample Detection Performance of R‐CHIP. a) Schematic diagram of the R‐CHIP detection process. b) Heatmap comparing 300 R‐CHIP test results from 100 clinical samples with results obtained from multiplex PCR (Samples in the red box represent false‐negative samples detected by R‐CHIP). c) ROC curve analysis of R‐CHIP detection for clinical samples, with AUC indicated by a 95% confidence interval. n = 200 (HPV‐16 = 100; HPV‐18 = 100). d, e) Grouped scatter plots illustrating the R‐CHIP detection results for HPV‐16 and HPV‐18 clinical samples. n = 100. f) Confusion matrix summarizing the performance of R‐CHIP in detecting clinical samples. g) Correlation comparison of detection values for clinical samples on and Off‐chip. Significance indicated: *P‐value < 0.05, **P‐value < 0.01, ***P‐value < 0.001, ****P‐value < 0.0001.

The clinical performance of microfluidic chips is critical to their market application and prospects. To meet the temperature control requirements for RPA‐CRISPR across various scenarios, this study surveyed multiple heating devices on the market and customized a rechargeable graphene fabric‐based smart temperature control device (Figure S11, Supporting Information). This device can precisely regulate temperatures between 32 and 60 °C and supports continuous operation for up to 72 hours, offering excellent portability and suitability for resource‐limited settings. Using this device as the heat source, we validated the practicality of the detection system by conducting multiplex HPV subtype analysis on 100 previously clinically analyzed cervical swab samples (Table S2, Supporting Information). Compared to traditional multiplex PCR methods that require approximately 6 hours, the introduced R‐CHIP employs an innovative spatial encoding strategy, enabling precise identification of multiple HPV subtypes using a single fluorescent dye (**Figure** **6**a). The entire detection process, including sample pre‐processing, RPA amplification, CRISPR reaction, and result readout, is shortened to about 50 minutes (Table S3, Supporting Information).

Figure 6b and Figure S12 (Supporting Information) visually demonstrate the detection results of clinical samples On‐chip, emphasizing the strong correlation between R‐CHIP and multiplex PCR. Compared to the Negative control (NTC), wells targeting HPV‐16 and HPV‐18 showed significantly higher fluorescence signals, indicating positivity for these subtypes. ROC curve analysis of 100 clinical samples further compared the detection performance of R‐CHIP with multiplex PCR (Figure 6c). The results showed that when the positivity cut‐off point for HPV‐16 and HPV‐18 were set at 627.55 and 615.27, respectively, the agreement between R‐CHIP and multiplex PCR reached 98% and 99%, demonstrating high consistency (Figure 6d–f; Table S4, Supporting Information). Despite some samples (HPV‐16 in samples #29 and #96, and HPV‐18 in sample #18) showing positive clinical results, their detection values in R‐CHIP were below the subtype‐specific Cut‐off point, leading to false‐negative results. Preliminary speculation suggests that this may be due to degradation of target DNA during storage and transportation or interference from the chip materials. Further analysis of the clinical PCR data revealed that these false‐negative samples exhibited relatively high CT values and low concentrations of the target analytes.

To determine whether the false‐negative results for positive samples were due to target DNA degradation or issues with the chip itself, this study re‐tested all clinical samples using an Off‐chip RPA‐CRISPR detection method (Figure S13 and Table S5, Supporting Information). The results demonstrate a high correlation between On‐chip and Off‐chip detection results, with R^2^ values of 0.95 and 0.93 for HPV‐16 and HPV‐18, respectively (Figure 6g). Similarly, HPV‐16 was not detected in samples #29 and #96, indicating that the target genes in positive samples are more likely to degrade during transportation or storage. Overall, the R‐CHIP platform demonstrated excellent subtype classification performance for these patient samples, with positive predictive values of 95.6% for HPV‐16 and 96.9% for HPV‐18, and negative predictive values of 100% for both subtypes. The overall agreement was 98% and 99%, respectively (Figure 6f). These findings underscore the high consistency between R‐CHIP and multiplex PCR detection methods. Compared to other existing detection methods, the R‐CHIP system demonstrates advantages including shorter turnaround time, ease of operation, and lower cost (Table S6, Supporting Information). These features make it especially suitable for community screening of HR‐HPV and large‐scale, rapid on‐site testing (Figure S14, Supporting Information).

### 2.8 Application of R‐CHIP Based on Deep Learning in HPV Clinical Analysis

**Figure 7:**
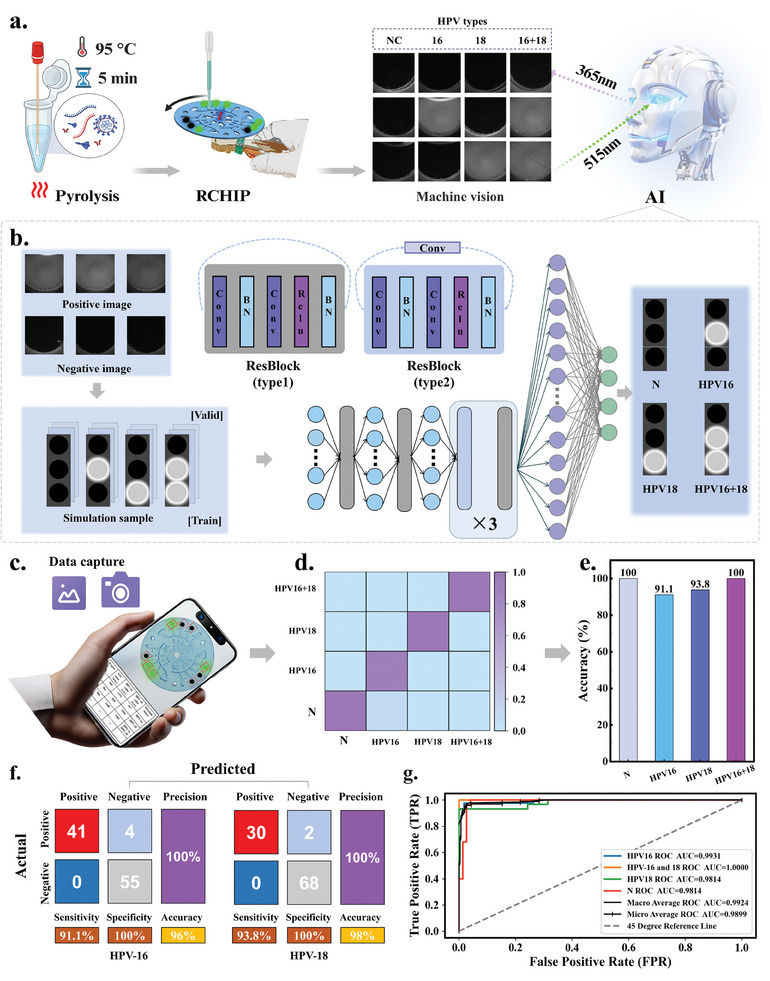
Application of R‐CHIP Based on Deep Learning in HPV Clinical Analysis. a) Schematic of the R‐CHIP Process for HR‐HPV Diagnosis. b) Dataset Construction Method and Image Classification Model Structure: Simulated images were obtained by merging positive and negative images with different fluorescence intensities and were divided into training and validation sets. The classic ResNet‐18 model was used to classify the results into N, HPV‐16, HPV‐18, and HPV‐16+18 categories (an additional 1×1 convolutional layer was used when the input and output channel numbers differed). c) Schematic diagram of a prediction done using the model deployed on a mobile phone. Direct reading of the result can be done by uploading a photo or taking a picture on mobile phone. d) Model predictions for 100 clinical samples. The model categorized the input images into four categories based on their characteristics: N, HPV‐16, HPV‐18, HPV‐16+18. e) Predictive accuracy of the model. The model's prediction accuracy for the four categories was 100%, 91.1%, 93.8%, and 100%, respectively. f) Confusion matrix of the model's predictions for HPV‐16 and HPV‐18, respectively. g) ResNet‐18 Classification ROC Curves and AUC. Plots of single‐class ROC curves and overall macro‐ and micro‐averaged ROC curves for multiclassification models.

While image processing software can distinguish between negative and positive results through fluorescence analysis, its reliance on region selection and more complex operational procedures limits detection efficiency and convenience. To address these limitations effectively, this study introduces a smartphone‐based microscopic imaging system integrated with deep learning to streamline the classification of clinical samples (**Figure** **7**a and Figure S15, Supporting Information).

By simulating positive and negative samples under varying fluorescence intensities, we constructed a training set of 10976 samples and a validation set of 4000 samples for model fine‐tuning (Supporting Information: Artificial intelligence models). Employing the classic ResNet‐18 pre‐trained model for image classification, we performed preliminary predictions on sample categories (Figure 7b). Post‐initial training, the model was deployed onto mobile devices for real‐time classification of uploaded or on‐site captured images (Figure 7c). In testing with 100 clinical samples, the model successfully categorized them into four classes: N, HPV‐16, HPV‐18, and HPV‐16+18, achieving an overall accuracy of 94.0%. The specific classification accuracies were as follows: N at 100%, HPV‐16 at 91.1%, HPV‐18 at 93.8%, and HPV‐16+18 at 100% (Figure 7d–f). Compared to the fluorescence imaging system, the smart microimaging system has a slightly lower overall coincidence rate due to the quality of the training set and data extraction. However, the coincidence rate of the smart microimaging system has been significantly higher than that required for clinical diagnosis and has significantly reduced the analysis time while improving the overall portability of the system. The ROC curve of the model demonstrates excellent AUC metrics, with individual AUC values for the four classes being 0.9931, 1.0000, 0.9814, and 0.9814, respectively. The macro‐average and micro‐average AUC values were 0.9924 and 0.9899 (Figure 7g), indicating superior discrimination ability. In summary, the ResNet‐18 model not only accelerates the analysis process but also facilitates efficient, automated analysis of large‐scale clinical samples, thereby enhancing diagnostic capabilities in resource‐limited settings.

## 3 Conclusion

This study developed a hand‐driven multi‐nucleic acid intelligent detection platform named R‐CHIP, which enables rapid molecular diagnostics from sample to result within one hour without the need for specialized technical personnel. R‐CHIP successfully integrates microfluidic technology, recombinase polymerase amplification (RPA), CRISPR‐based detection, and AI‐assisted analysis, achieving efficient and precise nucleic acid testing. Clinical evaluation results demonstrated that R‐CHIP can rapidly genotype and diagnose HR‐HPV, exhibiting outstanding diagnostic accuracy across 300 tests performed on 100 clinical samples. R‐CHIP significantly reduces reliance on complex laboratory equipment through the innovative design of passive and active valves, combined with hand‐driven portable devices that enable low‐speed sequential reagent release and simplified sample pre‐processing. The application of RPA‐CRISPR technology ensures high detection accuracy, while the modular design of R‐CHIP supports parallel processing of multiple samples on a single chip. Furthermore, the integration of AI detection models and a mobile intelligent microscopic imaging system further enhances detection efficiency and operational convenience. The flexible chamber configuration and preloaded target‐specific crRNA enhance the platform's customization capabilities, providing robust technical support for community screening. With its user‐friendly interface, broad application scope, and precise reagent release mechanism, R‐CHIP also demonstrates significant potential in biochemical analysis, immunoassays, and antimicrobial resistance evaluation.

Although preliminary results validate the promising application of AI in HPV detection, R‐CHIP still faces challenges in transitioning to a mature commercial detection system, primarily due to limitations in training dataset size, model structure optimization, and reagent storage stability. Future research could focus on expanding the diversity of sample sizes, exploring more advanced deep learning algorithms and evaluation metrics to further improve accuracy and stability, and developing reagent embedding and lyophilization technologies to enhance storage and transportation convenience. Additionally, the platform can be extended to broader applications in biochemistry, immunology, and molecular diagnostics, meeting diverse needs such as emergency triage for infectious diseases and rapid diagnostics in small medical institutions. The integration of AI‐driven data processing models and cloud technology could not only further improve the accuracy of detection results but also enable real‐time sharing of disease information, thereby strengthening the monitoring and response capabilities for public health events.

## 4 Experimental Section

### Reagents and Materials

This study utilized an RPA amplification kit, RPA fluorometric diagnostic kit, Exo probes and *Lachnospiraceae* bacterium ND2006 Cas12a (LbCas12a), both purchased from gendX (Suzhou, China). RPA primers, crRNA sequences, plasmids of the HPV L1 gene (for HPV 16 and HPV 18), and the fluorescent quenching probe TBA11‐FQ were synthesized by Sangon Biotech Co., Ltd. (Shanghai, China). Relevant sequence information was in Table S1 (Supporting Information). HPV standard cell lines were purchased from Jingliang Technology Co., Ltd. (Shenzhen, China). DNA agarose gel electrophoresis and nucleic acid gel staining reagents were obtained from Yeasen Biotechnology Co., Ltd. (Shanghai, China). The tachometer was purchased from Taobao Agilent Electronics store. The hand‐driven device was purchased from Taobao Xiaohai Toy Store.

### Fabrication of the Microfluidic Chip

The microfluidic chip used in this study was designed using AutoCAD software (Autodesk, California, USA) and exported as a.dxf file. It was then cut and manufactured using a CO_2_ laser cutting machine (Leishe Technology Co., Ltd., Shenzhen, China) and poly‐ (methyl methacrylate) (PMMA). The chip had a diameter of 10.5 cm and a thickness of 2.2 mm (Figure 2a). The chip consisted of a five‐layer structure, from top to bottom: a top cover (0.4 mm), a chamber layer (1.0 mm), an isolation layer (0.1 mm), a siphon valve layer (0.3 mm), and a bottom cover (0.4 mm). Double‐sided adhesive tape or polypropylene vinyl film was used for laminating and bonding between the layers. Finally, the formed chips underwent low‐temperature hot press bonding at 50 °C for 10 minutes using a hot press machine (Hengwei Precision Technology, Suzhou, China).

### Design of RPA Primers and crRNA Sequences

For the conserved regions of the L1 gene in HPV 16 and HPV 18, corresponding RPA amplification primers were designed. The primer design follows the guidelines provided in the TwistAmp RPA kit manual. The designs were optimized using tools such as DNAMAN (https://www.lynnon.com/) and CE Design (http://www.ce‐mark.com/ce‐design.htm). Simultaneously, based on the universal crRNA design principles for the CRISPR‐Cas12a system, specific crRNA sequences were designed targeting the L1 gene of HPV 16 and HPV 18. Detailed sequences of all primers, crRNAs, and target genes were provided in Table S1 (Supporting Information).

### Design of the CRISPR Cleavage System

The 20 µL CRISPR cleavage system On‐chip consists of the following components: NEB buffer (5 µL, containing 40 mM NaCl, 4 mM MgCl₂, 40 µg mL^−1^ BSA, pH 7.9), Cas12a (1 µL, 3 µM), crRNA (4 µL, 1 µM), TBA11 (FAM‐TBA11‐BHQ1, abbreviated as TBA11, 5 µL, 1 µM), and target DNA (5 µL, either plasmid or RPA product). This mixture was incubated at 40 °C for 20 minutes.

For the Off‐chip 30 µL CRISPR cleavage system, the amount of target DNA was adjusted to 7.5 µL, while the proportions of the other components remain unchanged.

### Design of the RPA Detection System

Following the standard operating instructions of the gendX RPA kit, single RPA amplification reactions for HPV 16 and HPV 18 were conducted. Each reaction system had a volume of 50 µL and included the following components: 20 µL of rehydration buffer, 2.5 µL of forward primer (10 µM), 2.5 µL of reverse primer (10 µM), 18 µL of ddH₂O, 5 µL of template DNA, and 2 µL of activator. The reaction mixture was thoroughly mixed and incubated at 40 °C for 20 minutes.

To prevent primer aggregation and ensure sufficient amplification products for each target sequence, the experiment prepared a primer pool with equimolar ratios of forward and reverse primers for HPV 16 and HPV 18 for multiplex RPA reactions. The multiplex RPA reaction system also had a volume of 50 µL, containing 20 µL of rehydration buffer, 1.25 µL each of forward and reverse primers for HPV 16 and HPV 18 (10 µM), 5 µL of template DNA, 2 µL of activator, and an appropriate volume of ddH₂O to adjust the final volume. The reaction conditions were the same: 40 °C for 20 minutes.

In the negative control group (NTC), the template DNA was replaced with an equal volume of ddH₂O. The RPA amplification products can be validated through subsequent analysis methods (such as electrophoresis, cleavage, etc.) or stored at ‐20 °C for future use.

### Agarose Gel Electrophoresis

To evaluate the specificity and amplification efficiency of the RPA reactions, the experiment analyzed the RPA amplification products using agarose gel electrophoresis (1% w/v, pre‐stained with GelRed). The amplification products were separated on the agarose gel at 100V/60 mA for 50 minutes. Subsequently, the gel images were obtained using an agarose gel imaging system (BioRad, Shanghai, China).

### Preparation of Clinical Samples

A total of 100 human cervical cell specimens were collected from Maternal and Child Health Hospital of Hubei Province for the study of HPV nucleic acid detection. This research has received approval from the Medical Ethics Committee of Hubei Maternal and Child Health Hospital (2023IECXM055). Prior to testing, the collected samples were screened for HPV infection status using multiplex PCR (Tellgen Corporation, Shanghai, China) in the clinical laboratory. To protect the privacy of the subjects, the samples were de‐identified before submission, ensuring no personal identification information was included. The collected samples were stored at ‐80 °C until use.

### Clinical Sample Detection Based on Multiplex PCR

Before testing, samples were screened for HPV infection using multiplex PCR (Tellgen Corporation, Shanghai, China) in the clinical laboratory. Briefly, HPV DNA released from the samples was amplified using biotin‐labeled primers in a multiplex PCR reaction. The amplification products were then hybridized to color‐coded microspheres coated with HPV subtype‐specific probes. The microspheres were incubated with streptavidin‐phycoerythrin (SA‐PE). After washing, the microspheres were read on the Luminex 200 system (Luminex Corporation, Texas, USA). HPV subtypes were identified based on the characteristic fluorescent dye carried by the microspheres.

### Optical Imaging System and Image Processing

Fluorescence detection within the chip was performed using the Fluorescence imaging system. Fluorescence signals in each reaction well of the chip were imaged under a 4×microscope. The imaging data were further analyzed using Image‐Pro Plus 6.0. To quantitatively measure the fluorescence value of each reaction well, the images were imported into the Image‐Pro Plus 6.0 software. The “Line Profile” option was selected to analyze the fluorescence intensity values of each reaction well.

In addition to On‐chip fluorescence detection, the experiment also employed a simple UV excitation method using a mobile phone camera to capture fluorescence images of the Off‐chip reaction system under dark conditions. The collected photos were subjected to grayscale analysis using Image‐J.

### Generation of Deep Learning Classification Models

By freely combining 13 images of varying fluorescence intensity for positive results and 13 images for negative results, 10976 (13×13×13×4) training set samples were generated. Similarly, 4000 (10×10×10×4) validation set samples were constructed using 10 images. After training for 20 epochs on a pre‐trained ResNet‐18 model, the final fully connected layer was fine‐tuned to accommodate the required number of classifications, resulting in a classification model. This model was used to predict the classification of 100 clinical samples.

### Statistical Analysis

Statistical analysis of the experimental data was performed using GraphPad Prism 9 (GraphPad Software Inc, USA) and SPSS (IBM Inc, USA). Standard deviation and mean values were calculated from at least three identical measurements. Clinical detection performance was analyzed using linear regression, t‐tests, and ROC curve analysis.

## Conflict of Interest

The authors declare no conflict of interest.

## Author Contributions

T.X., Y.Z., and S.L. contributed equally to this work. T.X., Z.Y., S.L., and Y.T. conceived and designed the project. T.X. and Z.Y. developed and optimized the detection system. T.X. and S.L. performed the theoretical calculations. P.C., T.X., and C.D. analyzed clinical samples and developed the Deep learning model for image analysis. D.C. was responsible for ethical approval and the collection of clinical vaginal swab samples. T.X., Z.Y., S.L., C.D., H.W., Y.Z., H.L., and D.L. conducted the laboratory‐related experimental research. T.X., S.L., P.C., B.L., and Y.T. edited the manuscript. P.C., B.L., and Y.T. provided the detection concept, writing guidance, project supervision, and financial support. All authors provided feedback and reviewed the manuscript.

## Supporting information



Supplemental Movie 1

Supplemental Movie 2

Supporting Information

## Data Availability

The data that support the findings of this study are available in the supplementary material of this article.
