# Supplementary material for: Deep Learning‐Enhanced Hand‐Driven Microfluidic Chip for Multiplexed Nucleic Acid Detection Based on RPA/CRISPR
Source: Adv Sci (Weinh). 2025 Mar 31;12(21):2414918. doi: 10.1002/advs.202414918 (PMC12140310; doi:10.1002/advs.202414918)
Supplement: Supplementary file 3 — Supporting Information [file ADVS-12-2414918-s003.pdf]

ADVANCED SCIENCE
Open Access

Supporting Information

for *Adv. Sci.*, DOI 10.1002/advs.202414918

Deep Learning-Enhanced Hand-Driven Microfluidic Chip for Multiplexed Nucleic Acid Detection Based on RPA/CRISPR

Tao Xu, Ying Zhang, Shunji Li, Chenxi Dai, Hongguo Wei, Dongjuan Chen, Yunjun Zhao, He Liu, Deliang Li, Peng Chen, Bi-Feng Liu* and Ye Tian**

Supporting Information

Deep Learning-enhanced Hand-driven Microfluidic Chip for Multiplexed Nucleic Acid Detection Based on RPA/CRISPR

Tao Xu[§,1,2], Ying Zhang[§,2], Shunji Li[§,2], Chenxi Dai[2], Hongguo Wei[1], Dongjuan Chen[3], Yunjun Zhao[1], He Liu[1], Deliang Li[1], Peng Chen[2,], Bi-Feng Liu[2,*] and Ye Tian[1,4,*]*

1 College of Medicine and Biological Information Engineering, Northeastern University Shenyang, 110169, China

2 The Key Laboratory for Biomedical Photonics of MOE at Wuhan National Laboratory for Optoelectronics-Hubei Bioinformatics & Molecular Imaging Key Laboratory, Systems Biology Theme, Department of Biomedical Engineering, College of Life Science and Technology, Huazhong University of Science and Technology, Wuhan, 430074, China

3 Department of Laboratory Medicine, Maternal and Child Health Hospital of Hubei Province, Tongji Medical College, Huazhong University of Science and Technology, Wuhan 430070, China.

4 Foshan Graduate School of Innovation, Northeastern University, Foshan, 528300, China.

§ These authors contributed equally to this work

* Corresponding authors

E-mail: tianye@bmie.neu.edu.cn (Lead contact); bfliu@mail.hust.edu.cn; gwchenpeng@mail.hust.edu.cn;

Table of Content

1. **Experimental Methods**

2. **Supplementary Figures**

Figure S1. R-CHIP operation process testing.

Figure S2. Accuracy and usability testing of R-CHIP manual centrifugation.

Figure S3. Overall operational workflow of the microfluidic chip demonstrated with dyes.

Figure S4. Off-chip HPV-16/18 non-amplification sensitivity detection.

Figure S5. RPA fluorescence assay for HR-HPV sensitivity detection.

Figure S6. Sensitivity of On-chip detection of different concentrations of HPV-16 and HPV-18 plasmids.

Figure S7. Off-chip evaluation of HPV-16 amplification sensitivity.

Figure S8. Off-chip evaluation of HPV-18 amplification sensitivity.

Figure S9. Gene sequence comparison and crRNA recognition site analysis of HPV subtypes.

Figure S10. The results of RPA amplification and CRISPR-specific cleavage in Off-chip HR-HPV detection system.

Figure S11. Smart temperature control device with charging capability.

Figure S12. Actual detection of clinical samples On-chip.

Figure S13. Comparative evaluation of clinical sample detection methods.

Figure S14. Presentation of current major methods for HPV molecular detection.

Figure S15. Smartphone-Based microscopic imaging device.

3. **Supplementary Tables**

Table S1. Sequence elements for RPA amplification and CRISPR-based detection systems.

Table S2. Clinical sample detection results from R-CHIP.

Table S3. Time required for complete detection workflow on the centrifugal microfluidic chip.

Table S4. Analysis of ROC curve results for clinical samples On-chip.

Table S5. Off-chip clinical sample detection outcomes.

Table S6. Various costs for a single test.

4. **Supplementary Movies**

Movie S1. The Principle of HR-HPV detection On-chip.

Movie S2. The operational process of R-CHIP.

5. **Artificial intelligence models**

1.Experimental Methods

Investigation of Chip Operation Protocol: To determine the minimum centrifugal terminal speed and acceleration required for successful operation of the microfluidic chip, this study employed a systematic approach. First, using our laboratory's centrifugal microfluidic video recording system, we captured the entire detection process of the chip at rotational speeds of 500 rpm, 1000 rpm, 1500 rpm, 2000 rpm, and 3000 rpm to identify the minimum terminal speed necessary for successful operation. Subsequently, to optimize centrifugal parameters, we tested the chip performance under various acceleration conditions (100 rpm/s, 250 rpm/s, 500 rpm/s, 750 rpm/s, 1000 rpm/s, 1500 rpm/s, and 2000 rpm/s) while maintaining a fixed terminal speed, to determine the minimum centrifugal acceleration required. To further validate the practical feasibility of these parameters, we measured the maximum rotational speed achievable within 5 seconds by 30 individuals from different professions (including 10 adult males and 20 adult females across various age groups) using a tachometer. This step aimed to evaluate the range of centrifugal forces that ordinary users can provide during actual operation, ensuring the user-friendliness and applicability of the microfluidic chip design.

Sample Processing: Following the manufacturer's instructions for the nucleic acid release reagent, the collected cervical swabs were placed in 1 mL of the reagent. The swabs were rotated and gently agitated. The tubes were then capped, inverted, and mixed 8-10 times. Subsequently, the samples were heated at 95°C using a heating module (such as a food heating pack or water bath) for 5 minutes to thoroughly lyse the cells and release intracellular DNA. The supernatant was collected for downstream detection. The lysate can be stored at room temperature (10-30°C) for up to 4 hours or at -20°C for up to 7 days.

RPA Fluorescence Method: Following the standard operating instructions of the gendX RPA fluorescence detection kit, we designed Exo probes specific for HPV-16 and HPV-18 and prepared the RPA amplification reaction mixtures (**Table S1**). Each 50 μL reaction mixture comprised: Premix (48 μL) (20 μL rehydration buffer, 2.1 μL forward primer (10 μM), 2.1 μL reverse primer (10 μM), 0.6 μL Exo probe (10 μM), and 18.2 μL ddH_2O; 5 μL template DNA) and 2 μL activator. First, the premix was prepared and thoroughly mixed, followed by a brief centrifugation. The premix was then transferred into tubes containing fluorescence amplification reagents, and different concentrations of HPV-16/18 plasmids were added to each tube. Finally, an additional 2 μL of activator was added to each tube, and the reactions were incubated at 40°C for 30 minutes.

For fluorescence detection using a PCR instrument, the reaction temperature was set to 40°C, and the detection program was initiated for 30 minutes, with FAM channel fluorescence values collected every 15 seconds. In the negative control group (NTC), template DNA was replaced with an equivalent volume of ddH_2O, while all other steps remained unchanged.

2.Supplementary Figures

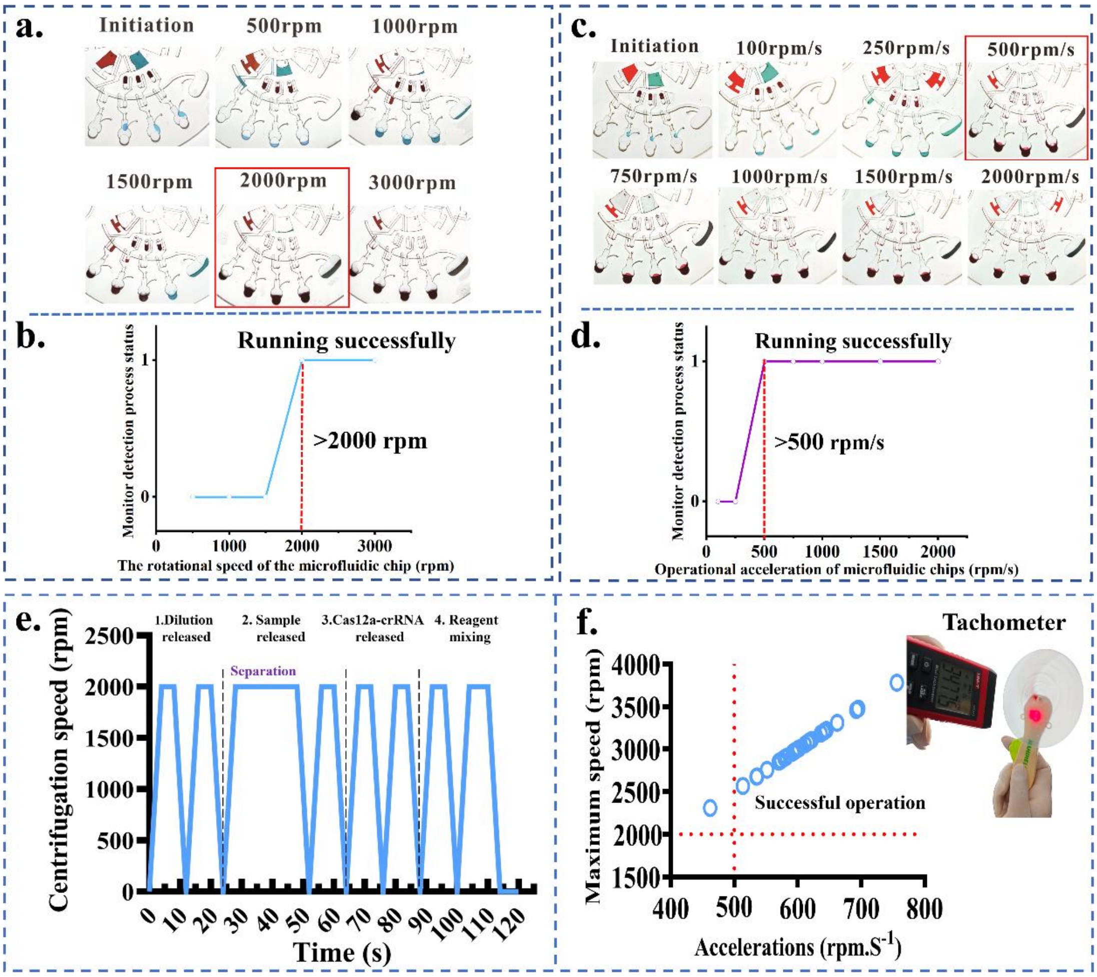

Figure S1. R-CHIP operation process testing. a) Physical demonstration of optimized minimum operating speed for the chip. b) Line chart of the chip's operating status（'0' indicates unsuccessful operation; '1' indicates successful completion of the detection process.）. c) Physical diagram of the chip's centrifugal acceleration optimization. d) Line chart illustrating the chip's operational status during acceleration optimization (The significance of '0' and '1' is illustrated in Figure b). e) Line diagram of the R-CHIP operation flow. f) Measurement of acceleration and maximum rotational speed in different populations using R-CHIP (n=30, including 10 adult males and 20 adult females, measuring the maximum rotational speed achievable within 5 seconds).

.

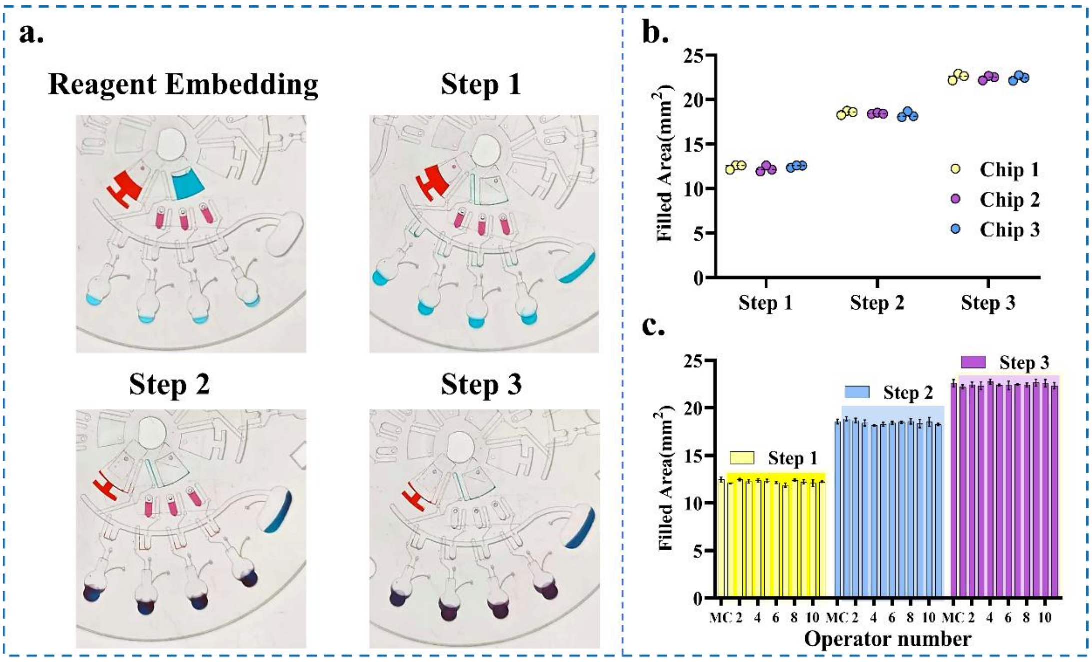

Figure S2. Accuracy and usability testing of R-CHIP manual centrifugation. a) Area changes in reaction chambers during liquid release stages under centrifugation. b) Intra-batch and inter-batch variability of the R-CHIP device. c) Comparison of reaction chamber areas for three-step procedures between manual and mechanical centrifugation for 11 participants ('MC' denotes area changes under centrifuge operation). n=3.

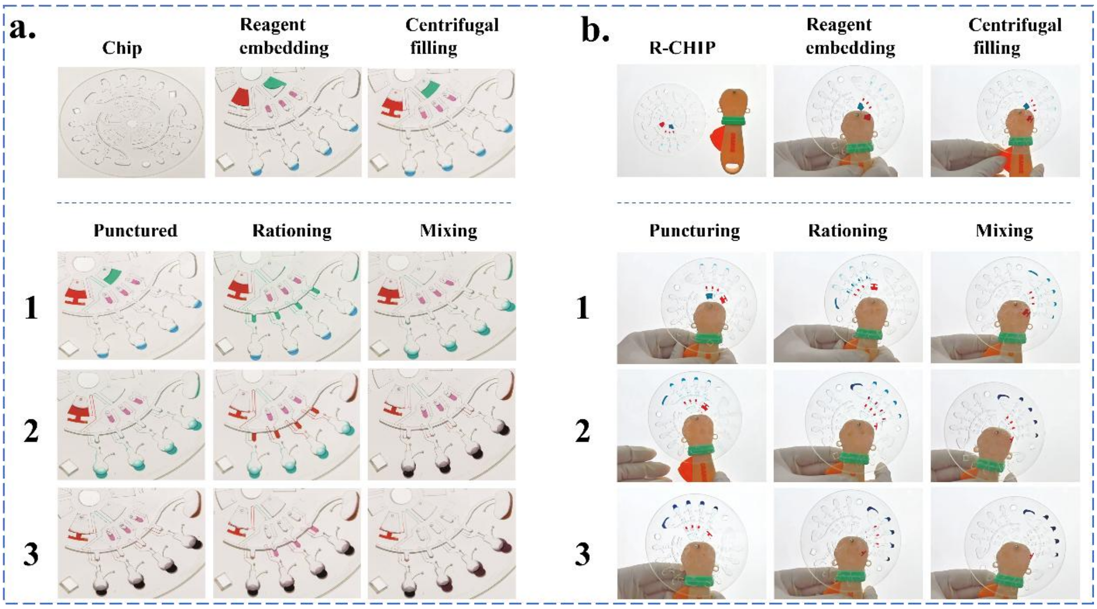

Figure S3. Overall operational workflow of the microfluidic chip demonstrated with dyes. a) Dye demonstration of the microfluidic chip under centrifuge operation (including: 1. Buffer chamber reagent addition process; 2. amplification sample addition process; 3. Cas12a-crRNA complex addition process.). b) Dye demonstration of the microfluidic chip under hand-driven operation, with reagent addition processes (1, 2, 3) identical to those in a).

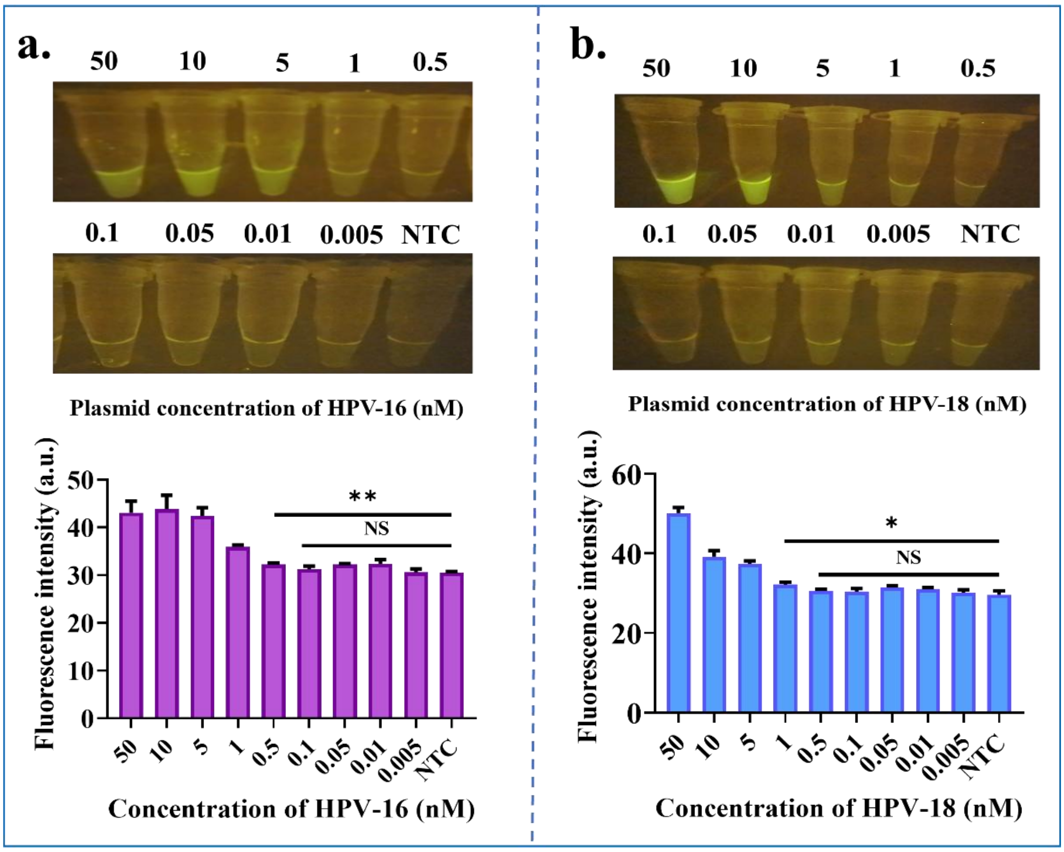

Figure S4. Off-chip HPV-16/18 non-amplification sensitivity detection. a) Non-amplification sensitivity of off-chip HPV-16. n=3. b) Non-amplification sensitivity of off-chip HPV-18. n=3. Significance indicated: *P-value < 0.05, **P-value < 0.01, ***P-value < 0.001, ****P-value < 0.0001.

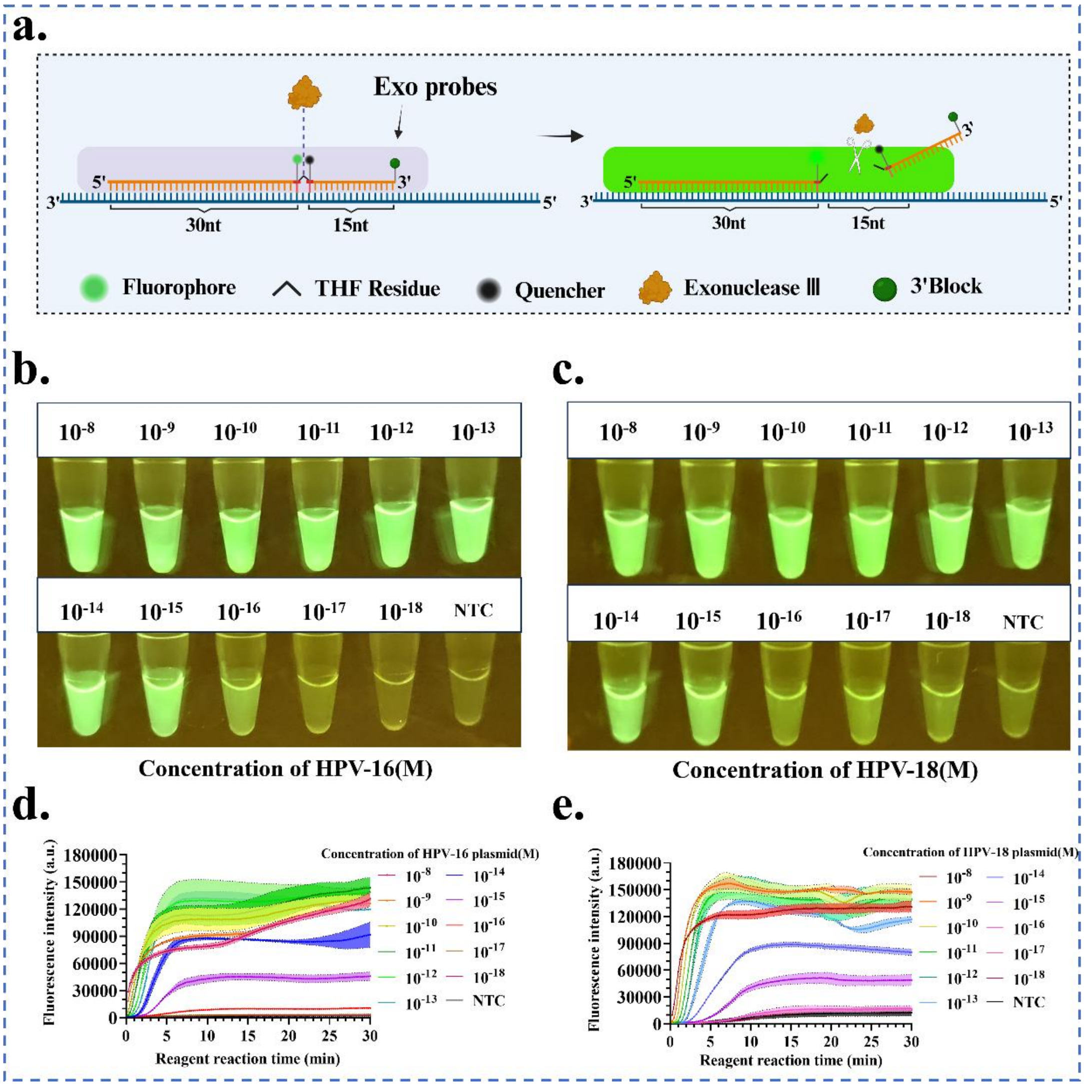

Figure S5. RPA fluorescence assay for HR-HPV sensitivity detection. a) Schematic of the RPA fluorescence detection principle. b, c) Fluorescence images showing sensitivity of RPA detection for HPV-16 and HPV-18. d, e) Validation of RPA isothermal amplification sensitivity for HPV-16 and HPV-18 using PCR instrument. n=3.

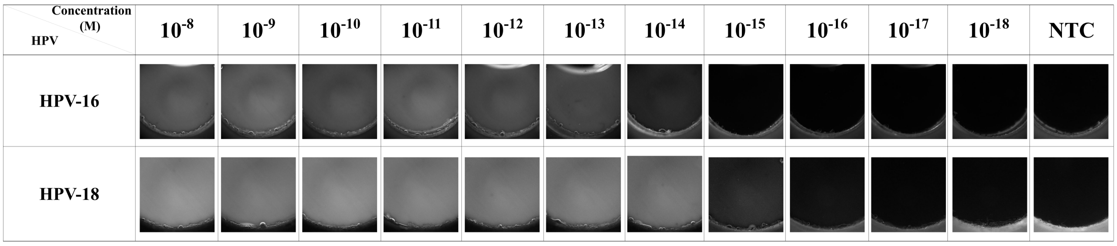

Figure S6. Sensitivity of On-chip detection of different concentrations of HPV-16 and HPV-18 plasmids.

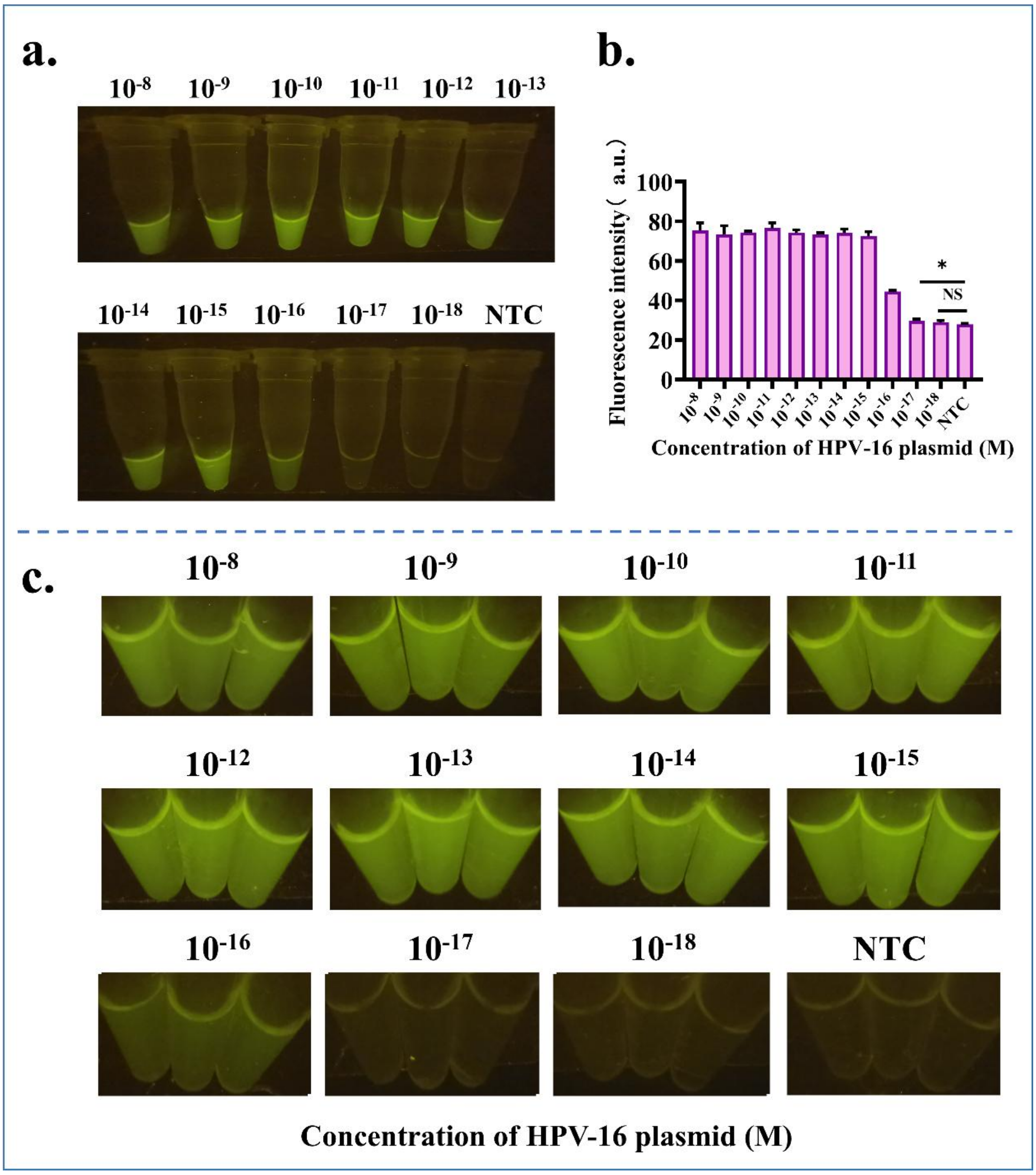

Figure S7. Off-chip evaluation of HPV-16 amplification sensitivity. a) Physical diagram of the sensitivity of CRISPR detection following HPV-16 RPA amplification conducted off-chip. b) Bar chart displaying the sensitivity of CRISPR detection subsequent to HPV-16 RPA amplification performed off-chip. n=3. c) Repeatability assessment of the sensitivity of CRISPR detection after HPV-16 RPA amplification, conducted off-chip. n=3.

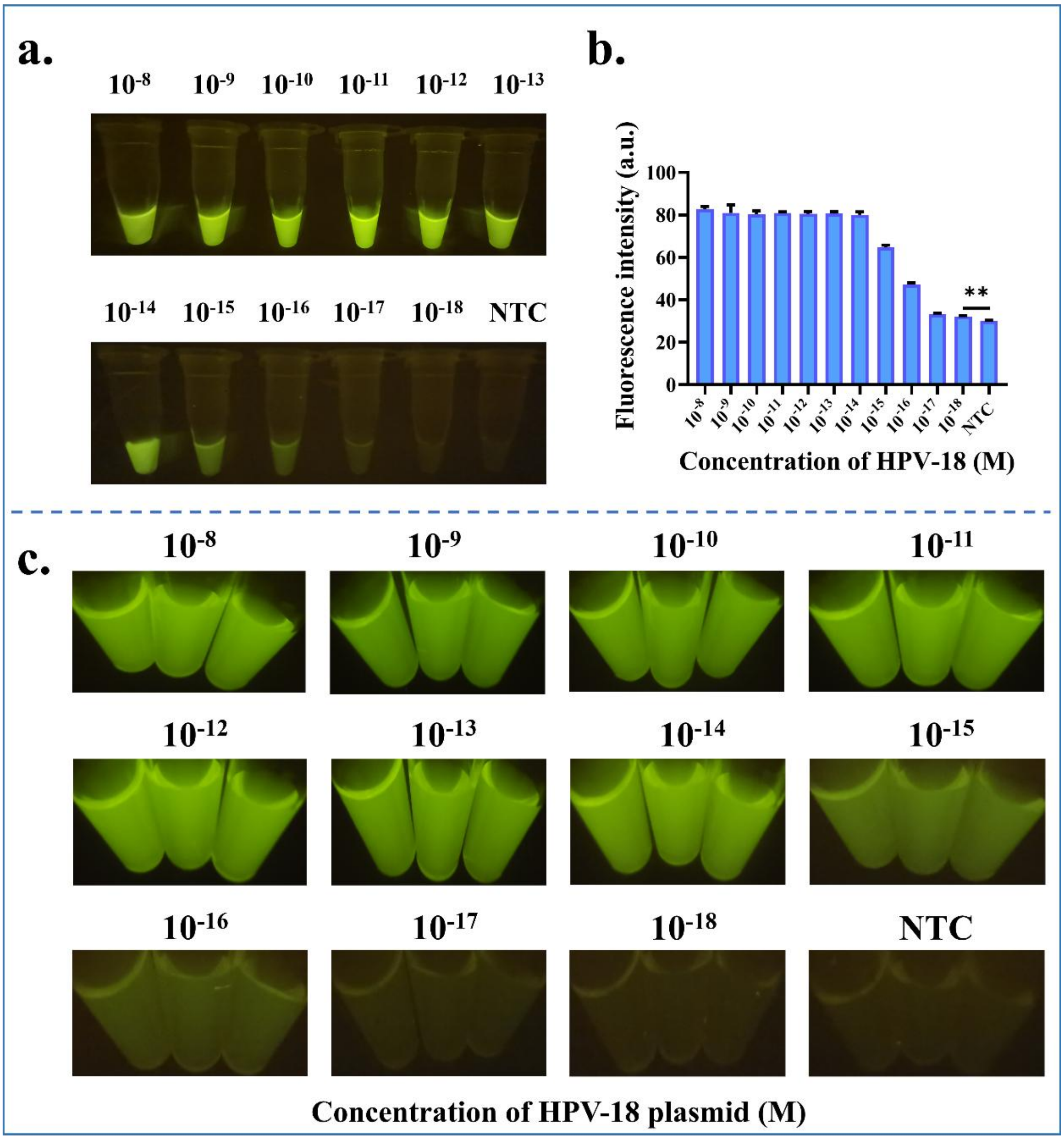

Figure S8. Off-chip evaluation of HPV-18 amplification sensitivity. a) Physical diagram of the sensitivity of CRISPR detection following HPV-18 RPA amplification conducted off-chip. b) Bar chart displaying the sensitivity of CRISPR detection subsequent to HPV-18 RPA amplification performed off-chip. n=3. c) Repeatability assessment of the sensitivity of CRISPR detection after HPV-18 RPA amplification, conducted off-chip. n=3.

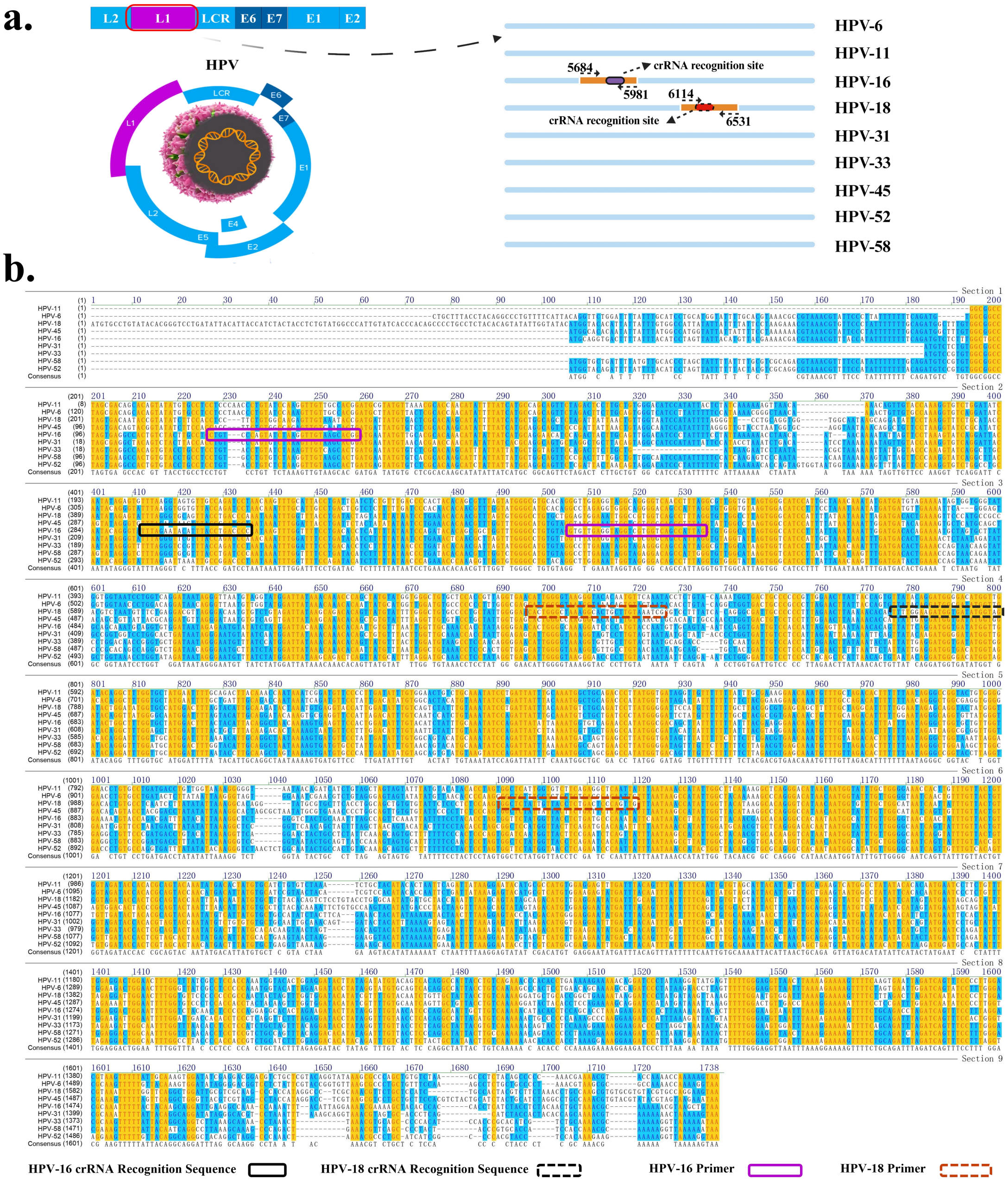

Figure S9. Gene sequence comparison and crRNA recognition site analysis of HPV subtypes. a) Amplified regions of the L1 gene and crRNA-specific recognition sites in HR-HPV. b) Sequence comparison of the L1 gene of the nine HPV subtypes and annotation of the gene amplification regions and crRNA recognition sites in HR-HPV.

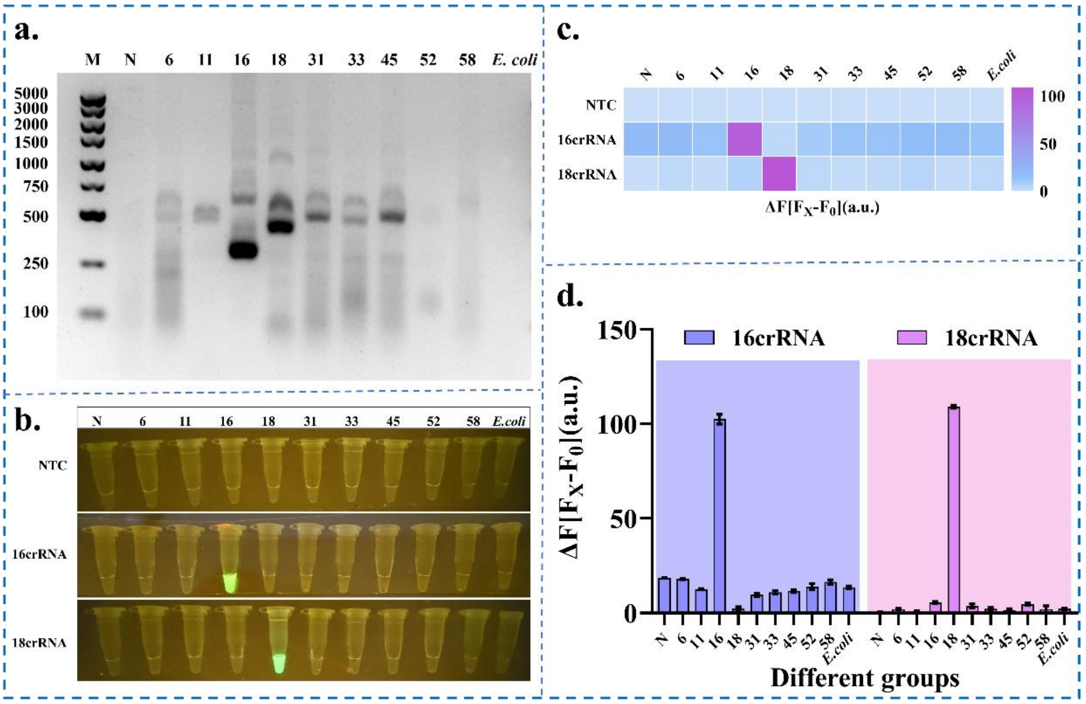

Figure S10. The results of RPA amplification and CRISPR-specific cleavage in the Off-chip HR-HPV detection system. a) Gel electrophoresis of multiplex RPA amplification products from 9 HPV subtypes and *E. coli*. b) Physical image of RPA amplification product after CRISPR-specific cleavage. c) Heatmap of data from RPA amplification products specifically cleaved by CRISPR. d) Bar graph of RPA amplification products specifically cleaved by CRISPR. n=3. (Note: Subjects included clinical samples of 9 HPV subtypes and *E. coli*, and all steps were done in the off-chip HR-HPV detection system.)

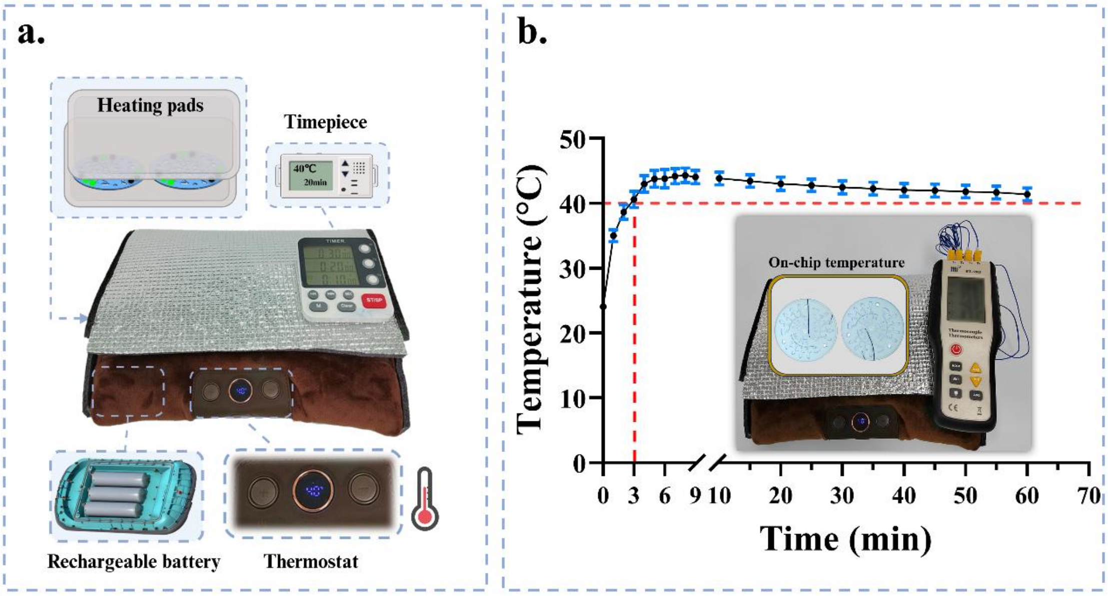

Figure S11. Smart temperature control device with charging capability. a) Schematic of the portable temperature control device, illustrating its heating function and component layout. b) Temperature dynamics within the chip reaction wells at a 40°C setpoint using the portable heater, showing the average temperature of four measurement points. n=4.

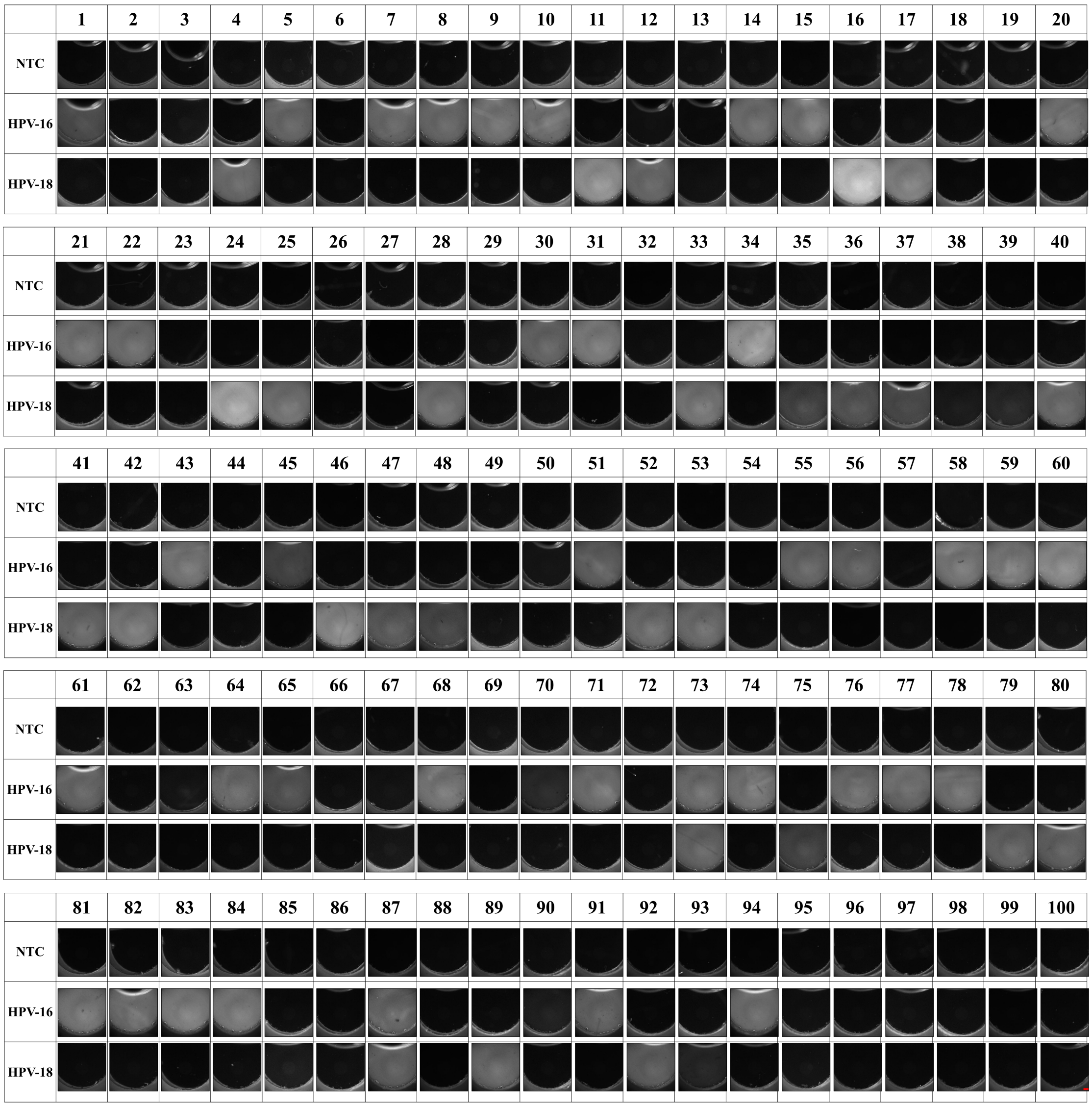

Figure S12. Actual detection of clinical samples On-chip. Column numbers represent samples 1 to 100, while the rows labeled NTC, HPV-16, and HPV-18 correspond to the detection targets for each well. n=100. Scale bar: 1cm.

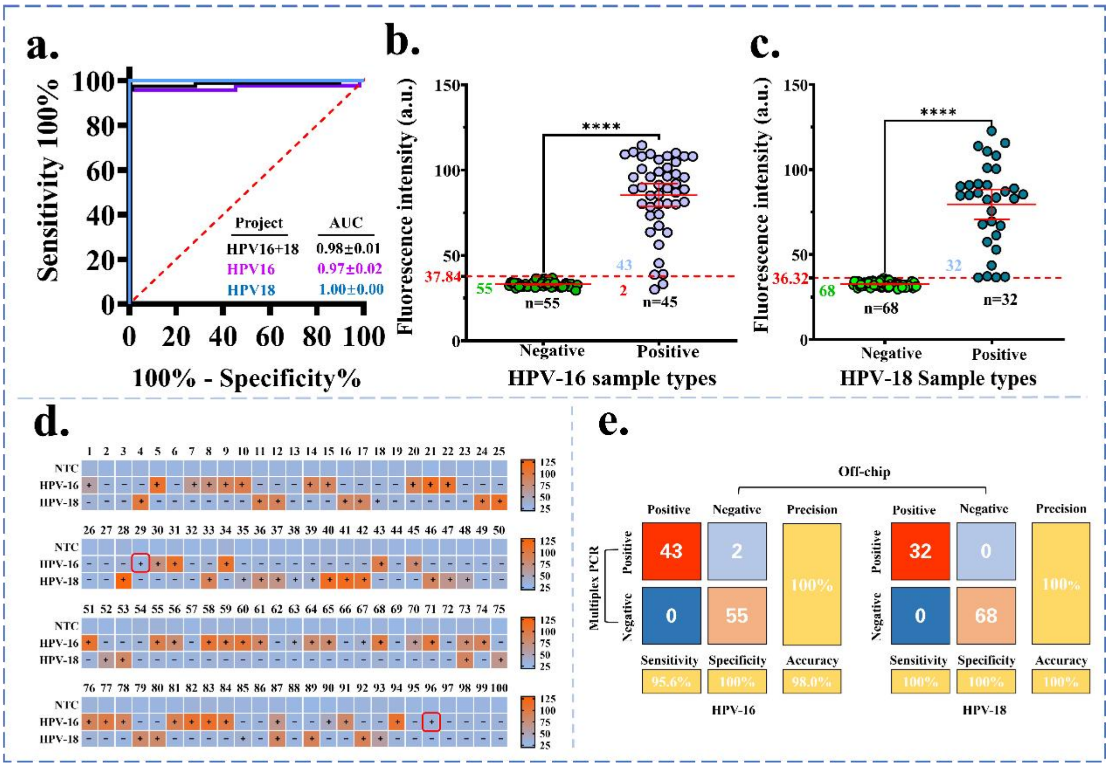

Figure S13. Comparative evaluation of clinical sample detection methods. a) ROC curve for the Off-chip detection of clinical samples, with AUC indicated by a 95% confidence interval. n=200 (HPV-16=100; HPV-18=100). b, c) Grouped scatter plot for the Off-chip detection of HPV-16 and HPV-18 clinical samples. n=100. d) Heatmap analysis for the Off-chip detection of clinical samples. e) Confusion matrix for Off-chip detection of clinical samples. Significance indicated: *P-value < 0.05, **P-value < 0.01, ***P-value < 0.001, ****P-value < 0.0001.

Methods			Comprehensive Performance				Time(min)	Cost		Clinical Samples
Methodologies	Amplification	Reporting method	LOD	Accuracy	Sample to answer	Multiplex	Assay time	Reagent	Homemade device	Number of tests
NATflow[1]	RPA	LFB	pM	●	●	●	45			55
CIALFB[2]	LAMP	Cas12a/LFB	3.1aM	●	●		90			14
CLIPON[3]	RPA	Cas12a/LFB	3.3 aM	●	●		120			6
M3-CRISPR[4]	RPA	Cas12a/LFB	1 aM	●	●		40			24
E-CRISPR[5]		Cas12a/ Electrochemistry	50pM		●		70			
AuNCs-CRISPR/ECL[6]		Cas12a/ ECL	0.48pM		●		70			Spiked sample
Enhanced Electrochemical CRISPR Biosensor[7]	RPA	Cas12a/ Electrochemistry	1pM	●	●		80			6
DAMR[8]	RPA	Cas12a/ Fluorescence	5aM	●	●	●	70			Spiked sample
DETECTR[9]	RPA	Cas12a/ Fluorescence	aM	●	●		70			25
PddCas[10]		Cas12a/ droplet digital	100aM	●	●		30			23
M-D3[11]	RPA	Cas12a/ droplet digital	1 aM	●	●	●	60			20
MiCaR[12]	RPA	Cas12a/ Fluorescence	1.7 fM	●	●	●	60			100
DROPT[13]	RPA	Cas12a/ Fluorescence	1 aM	●	●		40			60
CreDiT[14]	RPA	Cas12a/ Fluorescence	66zM	●	●		36			121
R-CASTI[15]	RPA	Cas12a/ Fluorescence	1 aM	●	●	●	80			21
**This work**	**RPA**	**Cas12a/AI**	**1 aM**	●	●	●	60			200

Figure S14. Presentation of current major methods for HPV molecular detection.

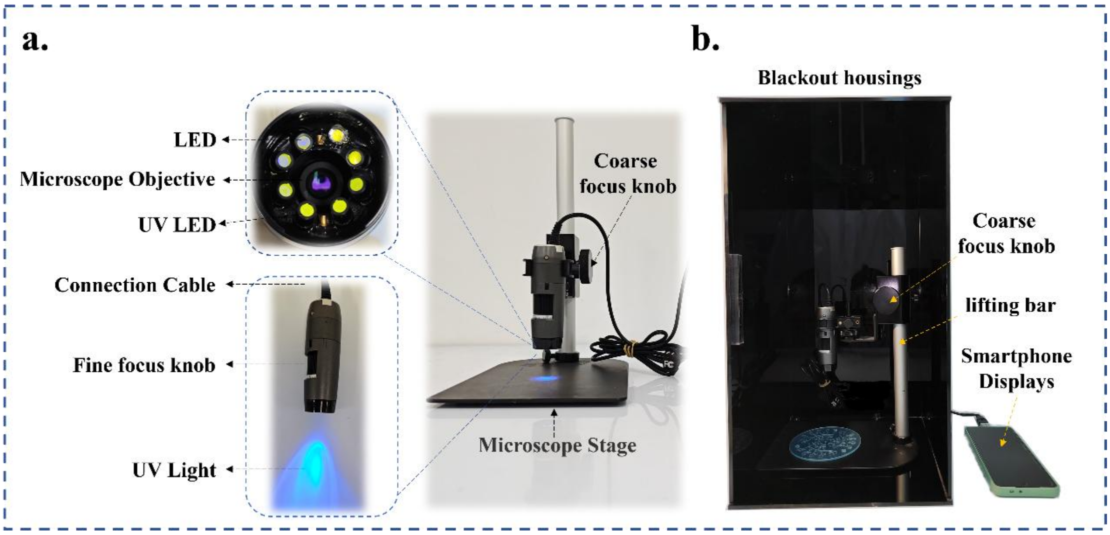

Figure S15. Smartphone-Based microscopic imaging device. a) Schematic of the components of the microscopic imaging system. b) Acquisition of images for R-CHIP AI detection using the smartphone-based microscopic imaging system.

3. Supplementary Tables

Table S1. Sequence elements for RPA amplification and CRISPR-based detection systems

Name	Sequence（5'-3'）	
Target HPV-16	AATACAGGGTATTTAGAATACATTTACCTGACCCCAATAAGTTTGGTTTTCCTGACAC	
Target HPV-18	TAAAAACACAGTTTTGGAAGATGGTGATATGGTAGATACTGGATATGGTGCCATGGA	
HPV-16 crRNA	UAAUUUCUACUAAGUGUAGAUGAAUACAUUUACCUGACCCC	
HPV-18 crRNA	UAAUUUCUACUAAGUGUAGAUGAAGAUGGUGAUAUGGUAGA	
plasmid vector	pUC19	
TBA11	6'FAM-GGTTGGTGTGG-BHQ1	
HPV-16 L1 gene	……ACTTGCCTCCTGTCCCAGTATCTAAGGTTGTAAGCACGGATGAATATGTTGCACGCACAAACATATATTATCATGCAGGAACATCCAGACTACTTGCAGTTGGACATCCCTATTTTCCTATTAAAAAACCTAACAATAACAAAATATTAGTTCCTAAAGTATCAGGATTACAATACAGGGTATTTAGAATACATTTACCTGACCCCAATAAGTTTGGTTTTCCTGACACCTCATTTTATAATCCAGATACACAGCGGCTGGTTTGGGCCTGTGTAGGTGTTGAGGTAGGTCGTGGTCAGCCATTAGGTG……	
HPV-18 L1 gene	……GGGAACACTGGGCTAAAGGCACTGCTTGTAAATCGCGTCCTTTATCACAGGGCGATTGCCCCCCTTTAGAACTTAAAAACACAGTTTTGGAAGATGGTGATATGGTAGATACTGGATATGGTGCCATGGACTTTAGTACATTGCAAGATACTAAATGTGAGGTACCATTGGATATTTGTCAGTCTATTTGTAAATATCCTGATTATTTACAAATGTCTGCAGATCCTTATGGGGATTCCATGTTTTTTTGCTTACGGCGTGAGCAGCTTTTTGCTAGGCATTTTTGGAATAGAGCAGGTACTATGGGTGACACTGTGCCTCAATCCTTATATATTAAAGGCACAGGTATGCGTGCTTCACCTGGCAGCTGTGTGTATTCTCCCTCTCCAAGTGGCTCTATTGTTACCTCTGACTCCCAGTTGTTTA……	
HPV-16 Exo Probe	TTTGGTTTTCCTGACACCTCATTTTATAA(FAM-dT)(THF)(BHQ1-dT)AGATACACAGCGGC(C3-SPACER)	
HPV-16 Primer	Pair 1	Reverse: AAAATAAACTGTAAATCATATTCCTC<br>Forward：GCACAGGGCCACAATAATGG
	Pair 2	Reverse: CACAACCGAAGCGTAGAGTCACACTTGCAA<br>Forward：CATGGAGATACACCTACATTGCATGAATAT
	Pair 3	Reverse: CTAATGGCTGACCACGACCTACCTCAACAC<br>Forward：CTGTCCCAGTATCTAAGGTTGTAAGCACGG
HPV-18 Exo Probe	CAGGTATGCGTGCTTCACCTGGCAGCTGTG(FAM-dT)(THF)(BHQ1-dT)ATTCTCCCTCTCC(C3-SPACER)	
HPV-18 Primer	Pair 1	Reverse： AAAATAAACTGCAAATCATATTCCTC<br>Forward：GCACAGGGTCATAACAATGG
	Pair 2	Reverse: TACTAGCTCAATTCTGGCTTCTTCACACTTACA<br>Forward：AAGACATTGTATTGCATTTAGACCCCAAA
	Pair 3	Reverse: CAACTGGGAGTCAGAGGTAACAATAGAGCC<br>Forward：CACTGGGCTAAAGGCACTGCTTGTAAATCG
* Green color represents the primer groups used in the multiplex detection of the R-CHIP.		

Table S2. Clinical sample detection results from R-CHIP

Sample No.	R-CHIP fluorescence value (a.u.)			Multiplex PCR		Sample No.	R-CHIP fluorescence value (a.u.)			Multiplex PCR	
	NTC	HPV16	HPV18	HPV16	HPV18		NTC	HPV16	HPV18	HPV16	HPV18
1	559.78	1045.49	569.97	1	0	51	559.90	1759.93	566.72	1	0
2	575.85	558.42	554.83	0	0	52	557.53	557.11	1162.90	0	1
3	558.12	564.40	569.44	0	0	53	555.25	556.30	1203.26	0	1
4	571.60	566.61	2192.27	0	1	54	557.92	555.49	564.22	0	0
5	578.85	1432.36	555.16	1	0	55	567.61	1400.20	572.62	1	0
6	580.06	573.06	588.66	0	0	56	567.51	1192.77	566.55	1	0
7	584.73	1230.56	549.52	1	0	57	560.62	562.96	566.08	0	0
8	578.21	1507.51	559.07	1	0	58	571.53	1545.56	568.25	1	0
9	579.07	1789.02	568.07	1	0	59	567.94	1524.86	563.22	1	0
10	589.23	1913.96	561.97	1	0	60	566.38	1625.68	562.31	1	0
11	584.79	597.66	1703.51	0	1	61	570.71	1287.51	564.65	1	0
12	580.21	591.04	1655.65	0	1	62	560.81	551.02	564.85	0	0
13	583.30	560.64	605.29	0	0	63	566.01	639.06	561.62	1	0
14	585.73	1647.86	576.22	1	0	64	560.09	1303.89	572.02	1	0
15	586.32	1527.76	582.40	1	0	65	567.66	1116.42	570.17	1	0
16	584.51	567.96	1657.01	0	1	66	560.02	569.71	563.89	0	0
17	588.31	572.03	1693.36	0	1	67	558.15	633.94	580.35	1	0
18	587.38	568.57	557.50	0	1	68	565.31	1524.64	567.57	1	0
19	581.82	564.26	533.88	0	0	69	566.67	557.70	564.55	0	0
20	585.34	1805.82	556.21	1	0	70	569.98	941.37	572.12	1	0
21	589.12	2004.85	550.35	1	0	71	570.34	1638.60	562.31	1	0
22	582.74	1960.92	547.58	1	0	72	560.60	569.33	564.92	0	0
23	581.57	571.63	551.96	0	0	73	560.09	1166.46	1218.37	1	1
24	563.59	570.11	1756.41	0	1	74	564.57	1391.97	563.53	1	0
25	565.85	569.47	1761.89	0	1	75	564.64	573.18	902.29	0	1
26	574.99	560.04	549.95	0	0	76	555.21	1402.53	573.01	1	0
27	571.12	556.58	554.87	0	0	77	566.63	1499.75	571.82	1	0
28	554.76	561.59	1753.30	0	1	78	568.22	1402.87	533.40	1	0
29	566.72	566.92	568.81	1	0	79	562.98	558.35	1245.35	0	1
30	562.45	1521.58	562.93	1	0	80	566.08	562.62	1157.48	0	1
31	566.29	2066.06	570.49	1	0	81	530.68	1441.46	570.84	1	0
32	561.94	569.01	562.82	0	0	82	558.71	1796.50	578.24	1	0
33	568.98	621.17	1633.16	0	1	83	563.08	1836.70	529.74	1	0
34	558.27	2018.70	563.00	1	0	84	555.09	1570.56	567.62	1	0
35	562.47	571.63	880.25	0	1	85	572.95	567.86	625.24	0	1
36	568.63	576.42	1043.51	0	1	86	567.53	573.99	570.24	0	0
37	557.35	581.25	910.34	0	1	87	567.08	1001.12	1227.31	1	1
38	561.24	568.35	734.85	0	1	88	571.38	565.23	577.70	0	0
39	550.15	602.42	774.02	0	1	89	583.92	565.38	1216.12	0	1
40	558.86	566.74	1786.82	0	1	90	583.72	719.17	569.63	1	0

41	548.39	563.11	1787.56	0	1	91	590.14	1216.02	567.84	1	0
42	565.92	564.81	1718.02	0	1	92	576.18	561.97	1328.04	0	1
43	570.20	1692.44	576.75	1	0	93	577.34	557.42	893.56	0	1
44	562.66	561.62	568.05	0	0	94	568.24	1862.66	569.86	1	0
45	550.37	1268.88	561.56	1	0	95	567.43	578.74	574.87	0	0
46	554.01	571.37	1673.42	0	1	96	569.79	574.47	563.89	1	0
47	563.33	572.15	1008.17	0	1	97	569.37	564.36	566.47	0	0
48	559.63	567.11	989.76	0	1	98	555.05	567.74	563.97	0	0
49	563.54	558.78	564.84	0	0	99	566.83	562.23	561.42	0	0
50	551.69	611.37	573.71	0	0	100	562.08	562.84	588.30	0	0

Table S3. Time required for complete detection workflow on the centrifugal microfluidic chip

Step	Operation	Reaction	Time
1	Sample lysis	Release intracellular DNA	About 5 min
2	RPA amplification	Amplify the target DNA	About 20 min
3	Chip operation	Reagent dispensing and sequential released	About 2 min
4	CRISPR reaction	Specific recognition and cleavage	About 20 min
5	Result detection	Fluorescence value detection	About 5 min

Table S4. Analysis of ROC curve results for clinical samples On-chip

HPV-16				HPV-18			
Cut-off point	Sensitivity	1-Specificity	Youden index	Cut-off point	Sensitivity	1-Specificity	Youden index
550.0200	1.000	1.000	0.000	528.7400	1.000	1.000	0.000
553.2550	1.000	0.982	0.018	531.5700	1.000	0.985	0.015
555.8950	1.000	0.964	0.036	533.6400	1.000	0.971	0.029
556.4400	1.000	0.945	0.055	540.7300	1.000	0.956	0.044
556.8450	1.000	0.927	0.073	548.5500	1.000	0.941	0.059
557.2650	1.000	0.909	0.091	549.7350	1.000	0.926	0.074
557.5600	1.000	0.891	0.109	550.1500	1.000	0.912	0.088
558.0250	1.000	0.873	0.127	551.1550	1.000	0.897	0.103
558.3850	1.000	0.855	0.145	553.3950	1.000	0.882	0.118
558.6000	1.000	0.836	0.164	554.8500	1.000	0.868	0.132
559.4100	1.000	0.818	0.182	555.0150	1.000	0.853	0.147
560.3400	1.000	0.800	0.200	555.6850	1.000	0.838	0.162
561.1150	1.000	0.782	0.218	556.8550	1.000	0.824	0.176
561.6050	1.000	0.764	0.236	558.2850	0.969	0.824	0.145

561.7950	1.000	0.745	0.255	560.2450	0.969	0.809	0.160
562.1000	1.000	0.727	0.273	561.4900	0.969	0.794	0.175
562.4250	1.000	0.709	0.291	561.5900	0.969	0.779	0.189
562.7300	1.000	0.691	0.309	561.7950	0.969	0.765	0.204
562.9000	1.000	0.673	0.327	562.1400	0.969	0.750	0.219
563.0350	1.000	0.655	0.345	562.5650	0.969	0.721	0.248
563.6850	1.000	0.636	0.364	562.8750	0.969	0.706	0.263
564.3100	1.000	0.618	0.382	562.9650	0.969	0.691	0.278
564.3800	1.000	0.600	0.400	563.1100	0.969	0.676	0.292
564.6050	1.000	0.582	0.418	563.3750	0.969	0.662	0.307
565.0200	1.000	0.564	0.436	563.7100	0.969	0.647	0.322
565.3050	1.000	0.545	0.455	563.9300	0.969	0.618	0.351
565.9950	1.000	0.527	0.473	564.0950	0.969	0.603	0.366
566.6750	1.000	0.509	0.491	564.3850	0.969	0.588	0.381
566.8300	1.000	0.491	0.509	564.6000	0.969	0.574	0.395
567.0150	0.978	0.491	0.487	564.7450	0.969	0.559	0.410
567.4250	0.978	0.473	0.505	564.8450	0.969	0.544	0.425
567.8000	0.978	0.455	0.523	564.8850	0.969	0.529	0.439
567.9100	0.978	0.436	0.541	565.5000	0.969	0.515	0.454
568.1550	0.978	0.418	0.560	566.2750	0.969	0.500	0.469
568.4600	0.978	0.400	0.578	566.5100	0.969	0.485	0.483
568.7900	0.978	0.382	0.596	566.6350	0.969	0.471	0.498
569.1700	0.978	0.364	0.614	567.1450	0.969	0.456	0.513
569.4000	0.978	0.345	0.632	567.5950	0.969	0.441	0.528
569.5900	0.978	0.327	0.651	567.7300	0.969	0.426	0.542
569.9100	0.978	0.309	0.669	567.9450	0.969	0.412	0.557
570.7400	0.978	0.291	0.687	568.0600	0.969	0.397	0.572
571.5000	0.978	0.273	0.705	568.1600	0.969	0.382	0.586
571.8300	0.978	0.236	0.741	568.5300	0.969	0.368	0.601
572.0900	0.978	0.218	0.760	569.1250	0.969	0.353	0.616
572.6050	0.978	0.200	0.778	569.5350	0.969	0.338	0.631
573.1200	0.978	0.182	0.796	569.7450	0.969	0.324	0.645
573.5850	0.978	0.164	0.814	569.9150	0.969	0.309	0.660
574.2300	0.978	0.145	0.832	570.0700	0.969	0.294	0.675
575.4450	0.956	0.145	0.810	570.2050	0.969	0.279	0.689
577.5800	0.956	0.127	0.828	570.3650	0.969	0.265	0.704
579.9950	0.956	0.109	0.846	570.6650	0.969	0.250	0.719
586.1450	0.956	0.091	0.865	571.3300	0.969	0.235	0.733
594.3500	0.956	0.073	0.883	571.9200	0.969	0.221	0.748
600.0400	0.956	0.055	0.901	572.0700	0.969	0.206	0.763
606.8950	0.956	0.036	0.919	572.3700	0.969	0.191	0.778
616.2700	0.956	0.018	0.937	572.8150	0.969	0.176	0.792
627.5550	0.956	0.000	0.956	573.3600	0.969	0.162	0.807
636.5000	0.933	0.000	0.933	574.2900	0.969	0.147	0.822

679.1150	0.911	0.000	0.911	575.5450	0.969	0.132	0.836
830.2700	0.889	0.000	0.889	576.4850	0.969	0.118	0.851
971.2450	0.867	0.000	0.867	577.2250	0.969	0.103	0.866
1023.3050	0.844	0.000	0.844	577.9700	0.969	0.088	0.881
1080.9550	0.822	0.000	0.822	579.2950	0.969	0.074	0.895
1141.4400	0.800	0.000	0.800	581.3750	0.969	0.059	0.910
1179.6150	0.778	0.000	0.778	585.3500	0.969	0.044	0.925
1204.3950	0.756	0.000	0.756	588.4800	0.969	0.029	0.939
1223.2900	0.733	0.000	0.733	596.9750	0.969	0.015	0.954
1249.7200	0.711	0.000	0.711	615.2650	0.969	0.000	0.969
1278.1950	0.689	0.000	0.689	680.0450	0.938	0.000	0.938
1295.7000	0.667	0.000	0.667	754.4350	0.906	0.000	0.906
1347.9300	0.644	0.000	0.644	827.1350	0.875	0.000	0.875
1396.0850	0.622	0.000	0.622	886.9050	0.844	0.000	0.844
1401.3650	0.600	0.000	0.600	897.9250	0.813	0.000	0.813
1402.7000	0.578	0.000	0.578	906.3150	0.781	0.000	0.781
1417.6150	0.556	0.000	0.556	950.0500	0.750	0.000	0.750
1436.9100	0.533	0.000	0.533	998.9650	0.719	0.000	0.719
1470.6050	0.511	0.000	0.511	1025.8400	0.688	0.000	0.688
1503.6300	0.489	0.000	0.489	1100.4950	0.656	0.000	0.656
1514.5450	0.467	0.000	0.467	1160.1900	0.625	0.000	0.625
1523.1100	0.444	0.000	0.444	1183.0800	0.594	0.000	0.594
1524.7500	0.422	0.000	0.422	1209.6900	0.563	0.000	0.563
1526.3100	0.400	0.000	0.400	1217.2450	0.531	0.000	0.531
1536.6600	0.378	0.000	0.378	1222.8400	0.500	0.000	0.500
1558.0600	0.356	0.000	0.356	1236.3300	0.469	0.000	0.469
1598.1200	0.333	0.000	0.333	1286.6950	0.438	0.000	0.438
1632.1400	0.311	0.000	0.311	1480.6000	0.406	0.000	0.406
1643.2300	0.289	0.000	0.289	1644.4050	0.375	0.000	0.375
1670.1500	0.267	0.000	0.267	1656.3300	0.344	0.000	0.344
1726.1850	0.244	0.000	0.244	1665.2150	0.313	0.000	0.313
1774.4750	0.222	0.000	0.222	1683.3900	0.281	0.000	0.281
1792.7600	0.200	0.000	0.200	1698.4350	0.250	0.000	0.250
1801.1600	0.178	0.000	0.178	1710.7650	0.219	0.000	0.219
1821.2600	0.156	0.000	0.156	1735.6600	0.188	0.000	0.188
1849.6800	0.133	0.000	0.133	1754.8550	0.156	0.000	0.156
1888.3100	0.111	0.000	0.111	1759.1500	0.125	0.000	0.125
1937.4400	0.089	0.000	0.089	1774.3550	0.094	0.000	0.094
1982.8850	0.067	0.000	0.067	1787.1900	0.063	0.000	0.063
2011.7750	0.044	0.000	0.044	1989.9150	0.031	0.000	0.031
2042.3800	0.022	0.000	0.022	2193.2700	0.000	0.000	0.000
2067.0600	0.000	0.000	0.000				

*** The cut-off points highlighted with a yellow background in the table represent the threshold values for each detection item.**

Table S5. Off-chip clinical sample detection outcomes

Sample No.	Off-chip value (a.u.)			Multiplex PCR		Sample No.	Off-chip value (a.u.)			Multiplex PCR	
	NTC	HPV16	HPV18	HPV16	HPV18		NTC	HPV16	HPV18	HPV16	HPV18
1	34.21	56.32	36.13	1	0	51	31.04	108.02	34.02	1	0
2	30.53	33.79	32.64	0	0	52	29.95	33.94	61.36	0	1
3	30.77	32.32	34.73	0	0	53	30.59	32.00	84.75	0	1
4	32.06	33.10	101.05	0	1	54	33.27	34.80	32.97	0	0
5	30.78	110.05	30.57	1	0	55	34.53	96.02	34.65	1	0
6	30.27	31.89	33.98	0	0	56	32.24	78.52	31.48	1	0
7	31.90	63.70	32.53	1	0	57	30.12	31.25	31.17	0	0
8	31.08	73.35	31.64	1	0	58	33.87	106.14	34.34	1	0
9	31.03	88.83	34.25	1	0	59	32.45	96.37	33.16	1	0
10	32.17	95.87	35.46	1	0	60	33.73	104.73	34.90	1	0
11	31.23	36.56	91.60	0	1	61	32.19	85.14	32.86	1	0
12	31.12	35.27	88.91	0	1	62	30.45	32.05	31.77	0	0
13	31.28	32.30	34.30	0	0	63	29.06	39.10	30.28	1	0
14	31.31	89.89	33.81	1	0	64	30.52	80.14	32.41	1	0
15	32.39	85.59	34.02	1	0	65	30.63	80.42	31.05	1	0
16	30.70	32.27	83.56	0	1	66	29.55	31.80	31.72	0	0
17	34.32	35.33	85.44	0	1	67	29.39	38.81	33.58	1	0
18	30.50	33.32	36.64	0	1	68	32.64	99.11	35.05	1	0
19	32.68	33.11	34.63	0	0	69	28.88	31.24	31.44	0	0
20	33.20	91.39	34.33	1	0	70	31.44	63.43	32.24	1	0
21	28.83	114.50	34.99	1	0	71	32.54	101.51	33.99	1	0
22	34.06	108.17	33.69	1	0	72	30.59	33.63	31.41	0	0
23	30.92	33.06	33.04	0	0	73	33.04	80.00	87.18	1	1
24	32.04	35.15	100.45	0	1	74	31.45	88.76	31.05	1	0
25	31.13	34.41	110.80	0	1	75	28.78	32.55	67.01	0	1
26	29.97	31.54	30.29	0	0	76	33.59	89.32	34.50	1	0
27	31.60	32.94	32.15	0	0	77	31.17	97.69	33.00	1	0
28	32.97	35.94	122.74	0	1	78	31.02	86.87	30.50	1	0
29	29.31	30.18	32.37	1	0	79	30.28	30.78	82.99	0	1
30	29.89	74.15	31.25	1	0	80	30.92	32.57	82.37	0	1
31	32.10	109.53	34.20	1	0	81	30.69	92.04	31.25	1	0
32	29.14	31.99	32.74	0	0	82	33.27	108.10	32.63	1	0
33	30.50	36.87	90.17	0	1	83	34.78	110.63	33.35	1	0
34	35.48	109.27	33.59	1	0	84	32.07	100.69	32.11	1	0
35	29.57	32.56	43.61	0	1	85	31.79	31.52	37.24	0	1
36	30.91	33.28	75.67	0	1	86	30.71	32.84	31.71	0	0
37	30.71	33.57	69.59	0	1	87	31.58	67.47	85.03	1	1
38	30.19	31.60	36.52	0	1	88	29.39	29.48	30.46	0	0
39	31.29	34.97	36.96	0	1	89	28.56	31.54	90.96	0	1
40	33.85	35.52	113.88	0	1	90	29.48	45.48	30.96	1	0

41	32.28	34.32	115.70	0	1	91	30.91	81.41	31.07	1	0
42	33.31	35.12	108.23	0	1	92	31.98	33.68	90.87	0	1
43	32.88	95.83	34.85	1	0	93	33.41	34.08	57.47	0	1
44	30.65	33.15	31.97	0	0	94	32.21	108.27	33.69	1	0
45	29.54	80.25	30.26	1	0	95	30.35	32.89	31.42	0	0
46	30.45	34.27	86.36	0	1	96	30.06	33.29	29.56	1	0
47	31.57	34.82	67.79	0	1	97	31.00	31.36	30.87	0	0
48	29.93	33.49	52.96	0	1	98	30.07	33.34	32.47	0	0
49	30.75	31.70	31.48	0	0	99	30.99	31.80	30.79	0	0
50	31.35	36.63	35.19	0	0	100	30.16	33.99	32.03	0	0

***In the table, a pink background indicates positive samples, while a yellow background denotes false-negative samples.**

Table S6. Various costs for a single test

Chip	Cost	Reagent	Cost
0.3 mm PMMA	0.20 CYN	Lysate	3.00 CNY
0.4 mm PMMA	0.27 CYN	RPA amplification	4.20 CNY
1.0 mm PMMA	0.24 CYN	LbCas12a	3.00 CNY
Polypropylene vinyl film	0.17 CYN	crRNA	0.15 CNY
Double-sided adhesive	0.06 CYN	NEB Buffer	0.01 CNY
-	-	ssDNA-FQ	0.08 CNY
Total	0.94 CYN	Total	10.44 CNY
Cost of testing per sample		11.38 CNY	

4. Supplementary Movies

Movie S1. The Principle of HR-HPV detection On-chip.

Movie S2. The operational process of R-CHIP.

5. Artificial intelligence models

(1). Environment and installation package (Python 3.10):

Package	Version	Package	Version
absl-py	1.4.0	google-pasta	0.2.0
aiohttp	3.9.5	grpcio	1.65.1
aiosignal	1.2.0	h5py	3.9.0
anyio	4.2.0	idna	3.4
argon2-cffi	21.3.0	ipykernel	6.28.0
argon2-cffi-bindings	21.2.0	ipython	8.20.0
asttokens	2.0.5	ipython-genutils	0.2.0
astunparse	1.6.3	jedi	0.19.1

async-lru	2.0.4	Jinja2	3.1.2
async-timeout	4.0.3	joblib	1.4.2
attrs	23.1.0	json5	0.9.6
Babel	2.11.0	jsonschema	4.19.2
beautifulsoup4	4.12.3	jsonschema-specifications	2023.7.1
bleach	4.1.0	jupyter_client	7.4.9
blinker	1.6.2	jupyter_core	5.7.2
brotlipy	0.7.0	jupyter-events	0.10.0
cachetools	5.3.3	jupyter-lsp	2.2.0
certifi	2024.8.30	jupyter_server	2.14.1
cffi	1.16.0	jupyter_server_terminals	0.4.4
charset-normalizer	3.3.2	jupyterlab	4.0.11
colorama	0.4.6	jupyterlab-pygments	0.1.2
comm	0.2.1	jupyterlab_server	2.25.1
contourpy	1.2.0	kiwisolver	1.4.4
cryptography	42.0.5	Markdown	3.6
cycler	0.11.0	MarkupSafe	2.1.3
Cython	3.0.11	matplotlib	3.7.2
debugpy	1.6.7	matplotlib-inline	0.1.6
decorator	5.1.1	mistune	2.0.4
defusedxml	0.7.1	mkl-fft	1.3.8
entrypoints	0.4	mkl-random	1.2.4
exceptiongroup	1.2.0	mkl-service	2.4.0
executing	0.8.3	mpmath	1.3.0
fastjsonschema	2.16.2	multidict	6.0.4
filelock	3.13.1	nb_conda	2.2.1
fonttools	4.51.0	nb-conda-kernels	2.3.1
frozenlist	1.4.0	nbclassic	1.1.0
fsspec	2024.9.0	nbclient	0.8.0
gast	0.4.0	nbconvert	7.10.0
gmpy2	2.1.2	nbformat	5.9.2
google-auth	2.22.0	nest-asyncio	1.6.0
networkx	3.1	rfc3986-validator	0.1.1
notebook	6.5.7	rpds-py	0.10.6
notebook_shim	0.2.3	rsa	4.7.2
numpy	1.24.3	scikit-learn	1.3.0
oauthlib	3.2.2	scipy	1.11.1
opencv-python	4.10.0.84	Send2Trash	1.8.2
overrides	7.4.0	setuptools	69.5.1

packaging	23.1	sip	6.7.12
pandocfilters	1.5.0	six	1.16.0
parso	0.8.3	sniffio	1.3.0
pillow	10.4.0	soupsieve	2.5
pip	24	stack-data	0.2.0
platformdirs	3.10.0	sympy	1.12
ply	3.11	tabulate	0.9.0
prometheus-client	0.14.1	tensorboard	2.17.0
prompt-toolkit	3.0.43	tensorboard-data-server	0.7.2
protobuf	4.25.3	tensorboard-plugin-wit	1.8.1
psutil	5.9.0	tensorboardX	2.2
pure-eval	0.2.2	terminado	0.17.1
pyasn1	0.4.8	threadpoolctl	3.5.0
pyasn1-modules	0.2.8	tinycss2	1.2.1
pycocotools	2.0.8	tomli	2.0.1
pycparser	2.21	torch	2.4.1
Pygments	2.15.1	torchaudio	2.4.1
PyJWT	2.8.0	torchvision	0.19.1
pyOpenSSL	24.0.0	tornado	6.4.1
pyparsing	3.0.9	tqdm	4.66.4
PyQt5	5.15.10	traitlets	5.14.3
PyQt5-sip	12.13.0	typing_extensions	4.12.2
PySocks	1.7.1	unicodedata2	15.1.0
python-dateutil	2.8.2	urllib3	1.26.16
python-json-logger	2.0.7	wcwidth	0.2.5
pytz	2024.1	webencodings	0.5.1
pywin32	305.1	websocket-client	1.8.0
pywinpty	2.0.10	Werkzeug	3.0.3
PyYAML	6.0.1	wheel	0.43.0
pyzmq	24.0.1	win-inet-pton	1.1.0
referencing	0.30.2	yarl	1.9.3
requests	2.31.0		
rfc3339-validator	0.1.4		

(2). Train (Jupyter lab)

```
import torch
import torch.nn as nn
import torch.optim as optim

import torchvision
from torchvision import datasets, models, transforms

import numpy as np
```

```python
import matplotlib.pyplot as plt

import time
import os

device = torch.device("cuda:0" if torch.cuda.is_available() else "cpu")
# device object

transforms_train = transforms.Compose([
    transforms.Resize((224, 224)),
    transforms.RandomHorizontalFlip(),  # data augmentation
    transforms.ToTensor(),
    transforms.Normalize([0.485, 0.456, 0.406], [0.229, 0.224, 0.225])
# normalization
])

transforms_val = transforms.Compose([
    transforms.Resize((224, 224)),
    transforms.ToTensor(),
    transforms.Normalize([0.485, 0.456, 0.406], [0.229, 0.224, 0.225])
])

data_dir = './datasets'
train_datasets = datasets.ImageFolder(os.path.join(data_dir, 'Train_1'),
transforms_train)
val_datasets = datasets.ImageFolder(os.path.join(data_dir, 'Valid'),
transforms_val)

train_dataloader = torch.utils.data.DataLoader(train_datasets,
batch_size=16, shuffle=True, num_workers=4)
val_dataloader = torch.utils.data.DataLoader(val_datasets,
batch_size=16, shuffle=True, num_workers=4)

print('Train dataset size:', len(train_datasets))
print('Validation dataset size:', len(val_datasets))

class_names = train_datasets.classes
print('Class names:', class_names)
plt.rcParams['figure.figsize'] = [12, 8]
plt.rcParams['figure.dpi'] = 60
plt.rcParams.update({'font.size': 20})


def imshow(input, title):
```

```
    # torch.Tensor to numpy
    input = input.numpy().transpose((1, 2, 0))
    # undo image normalization
    mean = np.array([0.485, 0.456, 0.406])
    std = np.array([0.229, 0.224, 0.225])
    input = std * input + mean
    input = np.clip(input, 0, 1)
    # display images
    plt.imshow(input)
    plt.title(title)
    plt.show()


# load a batch of train image
iterator = iter(train_dataloader)

# visualize a batch of train image
inputs, classes = next(iterator)
out = torchvision.utils.make_grid(inputs[:16])
imshow(out, title=[class_names[x] for x in classes[:16]])
model = models.resnet18(pretrained=True)
num_features = model.fc.in_features
model.fc = nn.Linear(num_features, 4)  # binary classification
(num_of_class == 4)
model = model.to(device)

criterion = nn.CrossEntropyLoss()
optimizer = optim.SGD(model.parameters(), lr=0.001, momentum=0.9)
num_epochs = 20
start_time = time.time()

for epoch in range(num_epochs):
    """ Training  """
    model.train()

    running_loss = 0.
    running_corrects = 0

    # load a batch data of images
    for i, (inputs, labels) in enumerate(train_dataloader):
        inputs = inputs.to(device)
        labels = labels.to(device)

        optimizer.zero_grad()
```

```
        outputs = model(inputs)
        _, preds = torch.max(outputs, 1)
        loss = criterion(outputs, labels)

        # get loss value and update the network weights
        loss.backward()
        optimizer.step()

        running_loss += loss.item() * inputs.size(0)
        running_corrects += torch.sum(preds == labels.data)

    epoch_loss = running_loss / len(train_datasets)
    epoch_acc = running_corrects / len(train_datasets) * 100.
    print('[Train #{}] Loss: {:.4f} Acc: {:.4f}% Time:
{:.4f}s'.format(epoch, epoch_loss, epoch_acc,
         time.time() - start_time))

    """ Validation"""
    model.eval()

    with torch.no_grad():
        running_loss = 0.
        running_corrects = 0

        for inputs, labels in val_dataloader:
            inputs = inputs.to(device)
            labels = labels.to(device)

            outputs = model(inputs)
            _, preds = torch.max(outputs, 1)
            loss = criterion(outputs, labels)

            running_loss += loss.item() * inputs.size(0)
            running_corrects += torch.sum(preds == labels.data)

        epoch_loss = running_loss / len(val_datasets)
        epoch_acc = running_corrects / len(val_datasets) * 100.
        print('[Validation #{}] Loss: {:.4f} Acc: {:.4f}% Time:
{:.4f}s'.format(epoch, epoch_loss, epoch_acc,

time.time() - start_time))
torch.save(model.state_dict(), "Model/HPV_cog_Model_1.pth")
print("That's it!")
```

(3). Prediction

```
import torch
import torch.nn as nn
from sklearn import metrics
from sklearn.metrics import roc_curve, auc
from sklearn.preprocessing import label_binarize
import numpy as np
from torchvision import datasets, models, transforms
import matplotlib.pyplot as plt

transforms_val = transforms.Compose([
    transforms.Resize((224, 224)),
    transforms.ToTensor(),
    transforms.Normalize([0.485, 0.456, 0.406], [0.229, 0.224, 0.225])
])
device = torch.device('cpu')
Pre_datasets = datasets.ImageFolder('Valid', transforms_val)
Pre_dataloader = torch.utils.data.DataLoader(Pre_datasets,
batch_size=16, shuffle=False, num_workers=0)
print('Validation dataset size:', len(Pre_datasets))
class_names = Pre_datasets.classes
print('Class names:', class_names)
model = models.resnet18(pretrained=True)
num_features = model.fc.in_features
model.fc = nn.Linear(num_features, 4) # binary classification
(num_of_class == 4)
model.load_state_dict(torch.load("Model/HPV_cog_Model_1.pth",
map_location=device))
criterion = nn.CrossEntropyLoss()
model.to(device)
model.eval()
scores = []
preds = []
label_s = []
with torch.no_grad():
    running_loss = 0.
    running_corrects = 0
    for inputs, labels in Pre_dataloader:
        inputs = inputs.to(device)
        label = labels.to(device)
        outputs = model(inputs)
        _, pred = torch.max(outputs, 1)
        loss = criterion(outputs, labels)
        running_loss += loss.item() * inputs.size(0)
```

```
        running_corrects += torch.sum(pred == labels.data)
        print(label)
        print(pred)
        label = label.tolist()
        pred = pred.tolist()
        outputs = outputs.tolist()
        label_s += label
        preds += pred
        scores += outputs
    loss = running_loss / len(Pre_datasets)
    acc = running_corrects / len(Pre_datasets) * 100.
    print('Loss: {:.4f} Acc: {:.4f}% '.format(loss, acc))
scores = np.array(scores)
print(scores[:, 1])
# Convert y tags into one-hot form
label_s = label_binarize(label_s, classes=[0, 1, 2, 3])
# 宏平均法计算 AUC
AUC = {}
FPR = {}
TPR = {}

for i in range(4):
    FPR[i], TPR[i], thresholds = roc_curve(label_s[:, i], scores[:, i])
    AUC[i] = auc(FPR[i],TPR[i])
print(AUC)

# Merge all FPRs and sort for de-emphasis
FPR_final = np.unique(np.concatenate([FPR[i] for i in
range(label_s.shape[1])]))
print(FPR_final)
# Calculating Macro-Average TPR
TPR_all = np.zeros_like(FPR_final)
for i in range(label_s.shape[1]):
    TPR_all += np.interp(FPR_final, FPR[i], TPR[i])
TPR_final = TPR_all / label_s.shape[1]
print(TPR_final)
# Calculate the final macro average AUC
AUC_final = auc(FPR_final, TPR_final)
print(f"Macro Average AUC with Random Forest: {AUC_final}")

# Leveling the predicted probability of each sample classification
obtained from labeling, and modeling
micro_labels = np.ravel(label_s)
micro_labels_pre = np.ravel(scores)
```

```
# Calculation of FPR, TPR under the micro-averaging method
micro_FPR, micro_TPR, mic_thresholds = metrics.roc_curve(micro_labels,
micro_labels_pre)
micro_AUC = metrics.auc(micro_FPR, micro_TPR)

# plot
plt.rc('font',family='Times New Roman')
plt.figure(figsize=(10, 5), dpi=250)
ax = plt.gca()  # 获取边框

ax.spines['bottom'].set_linewidth(2)
ax.spines['left'].set_linewidth(2)
ax.spines['top'].set_linewidth(2)
ax.spines['right'].set_linewidth(2)

# 使用不同的颜色和线型
plt.plot(FPR[0], TPR[0], color='#1f77b4', linestyle='-', label='HPV16
ROC  AUC={:.4f}'.format(AUC[0]), lw=2)
plt.plot(FPR[1], TPR[1], color='#ff7f0e', linestyle='-', label='HPV-16
and 18 ROC  AUC={:.4f}'.format(AUC[1]), lw=2)
plt.plot(FPR[2], TPR[2], color='#2ca02c', linestyle='-', label='HPV18
ROC  AUC={:.4f}'.format(AUC[2]), lw=2)
plt.plot(FPR[3], TPR[3], color='#FF3D24', linestyle='-', label='N ROC
AUC={:.4f}'.format(AUC[2]), lw=2)
# 宏平均 ROC 曲线
plt.plot(FPR_final, TPR_final, color='#000000', linestyle='-',
        label='Macro Average ROC  AUC={:.4f}'.format(AUC_final), lw=1.5)
# 45 度参考线
plt.plot(micro_FPR, micro_TPR, 'k|-', label='Micro Average ROC
AUC={:.4f}'.format(micro_AUC), lw=1.5)
plt.plot([0, 1], [0, 1], color='gray', linestyle='--', lw=2, label='45
Degree Reference Line')
plt.xlabel('False Positive Rate (FPR)', fontweight='bold', fontsize=16)
plt.ylabel('True Positive Rate (TPR)', fontweight='bold', fontsize=16)
# plt.title('ResNet-18 Classification ROC Curves and AUC', fontsize=18)
# plt.grid(linestyle='--', alpha=0.5)
plt.legend(loc='lower right', prop={'weight': 'bold'}, framealpha=0.9,
fontsize=12)
plt.xticks(fontsize=14, fontweight='bold')
plt.yticks(fontsize=14, fontweight='bold')
plt.show()
```

References

[1] K. A. Kundrod, M. Barra, A. Wilkinson, C. A. Smith, M. E. Natoli, M. M. Chang, J. B. Coole, A. Santhanaraj, C. Lorenzoni, C. Mavume, H. Atif, J. R. Montealegre, M. E. Scheurer, P. E. Castle, K. M. Schmeler, R. R. Richards-Kortum, *Sci Transl Med* **2023**, *15*, eabn4768.

[2] O. Mukama, T. Yuan, Z. He, Z. Li, J. d. D. Habimana, M. Hussain, W. Li, Z. Yi, Q. Liang, L. Zeng, *Sensors and Actuators B: Chemical* **2020**, *316*, 128119.

[3] Y. Tang, L. Qi, Y. Liu, L. Guo, R. Zhao, M. Yang, Y. Du, B. Li, *Angewandte Chemie International Edition* **2022**, *61*, e202115907.

[4] H. Zhou, Z. Xu, L. He, Z. Wang, T. Zhang, T. Hu, F. Huang, D. Chen, Y. Li, Y. Yang, X. Huang, *Anal Chem* **2023**, *95*, 3379.

[5] Y. Dai, R. A. Somoza, L. Wang, J. F. Welter, Y. Li, A. I. Caplan, C. C. Liu, *Angewandte Chemie International Edition* **2019**, *58*, 17399.

[6] P.-F. Liu, K.-R. Zhao, Z.-J. Liu, L. Wang, S.-Y. Ye, G.-X. Liang, *Biosensors and Bioelectronics* **2021**, *176*, 112954.

[7] Z. Li, X. Ding, K. Yin, Z. Xu, K. Cooper, C. Liu, *Biosensors and Bioelectronics* **2021**, *192*, 113498.

[8] K. Yin, X. Ding, Z. Li, H. Zhao, K. Cooper, C. Liu, *Analytical Chemistry* **2020**, *92*, 8561.

[9] J. S. Chen, E. Ma, L. B. Harrington, M. Da Costa, X. Tian, J. M. Palefsky, J. A. Doudna, *Science* **2018**, *360*, 436.

[10] Y. Xue, X. Luo, W. Xu, K. Wang, M. Wu, L. Chen, G. Yang, K. Ma, M. Yao, Q. Zhou, Q. Lv, X. Li, J. Zhou, J. Wang, *Analytical Chemistry* **2023**, *95*, 966.

[11] Y. Zhao, D. Chen, Z. Xu, T. Li, J. Zhu, R. Hu, G. Xu, Y. Li, Y. Yang, M. Liu, *Analytical Chemistry* **2023**, *95*, 3476.

[12] Z. Xu, D. Chen, T. Li, J. Yan, J. Zhu, T. He, R. Hu, Y. Li, Y. Yang, M. Liu, *Nature Communications* **2022**, *13*, 6480.

[13] Y. Cai, L. Zhuang, J. Yu, L. He, Z. Wang, T. Hu, L. Li, X. Li, H. Zhou, X. Huang, *Sensors and Actuators B: Chemical* **2024**, *405*, 135295.

[14] C. Y. Lee, H. Kim, I. Degani, H. Lee, A. Sandoval, Y. Nam, M. Pascavis, H. G. Park, T. Randall, A. Ly, C. M. Castro, H. Lee, *Nature Communications* **2024**, *15*, 6271.

[15] J. Han, J. Shin, E. S. Lee, B. S. Cha, S. Kim, Y. Jang, S. Kim, K. S. Park, *Biosensors and Bioelectronics* **2023**, *232*, 115323.
